# The Big Five Functional Levels of Vascular Calcification

**DOI:** 10.3390/biology15191708

**Published:** 2026-09-25

**Authors:** Ricardo Villa-Bellosta

**Affiliations:** 1 Department of Biochemistry and Molecular Biology, University of Santiago de Compostela, 15782 Santiago de Compostela, Spain; ricardo.villa@usc.es; 2 Health Research Institute of Santiago de Compostela (IDIS), Travesia da Choupana S/N, 15706 Santiago de Compostela, Spain

**Keywords:** vascular calcification, calcium phosphate, pyrophosphate, vascular smooth muscle cells, extracellular vesicles, calciprotein particles, cellular senescence, osteogenic reprogramming

## Abstract

Vascular calcification is often described either as passive deposition of calcium-phosphate crystals or as an active osteogenic process. Neither view alone explains how mineral formation begins, progresses and becomes self-sustaining within the vascular wall. Here, we organize the main mechanisms of vascular calcification into five functional levels according to their predominant causal role. Level I defines mineral permissiveness through calcium-phosphate pressure and extracellular mineralization inhibitors. Level II determines how nascent mineral is packaged, nucleated and retained through calciprotein particles, extracellular vesicles and extracellular matrix remodeling. Level III describes the cellular response, including vascular smooth muscle cell plasticity, BMP–WNT signaling and endothelial-to-mesenchymal transition. Level IV comprises inflammatory, oxidative, mitochondrial, senescent and epigenetic–metabolic mechanisms that amplify and stabilize calcification-prone states. Level V determines whether cellular stress can still be contained or progresses to proteostatic failure and regulated cell death. These levels are functionally coupled rather than sequential: mineral can alter cellular phenotype, while cellular dysfunction feeds back to weaken mineral inhibition and generate new nucleation surfaces. This framework places failure of mineral containment, rather than any single signaling pathway, at the center of vascular calcification and provides a basis for considering causal order and therapeutic timing across different disease settings.

## Simple Summary

Vascular calcification is the accumulation of calcium-phosphate mineral in the walls of blood vessels. It is frequently seen with aging and in cardiovascular and kidney disease, but no single mechanism explains how it develops. Mineral chemistry, changes in the vessel wall and the response of vascular cells all contribute. This review brings these different processes together to show how they interact and why calcification can progress in different ways depending on the disease setting. Viewing the field in this way may make it easier to compare experimental findings and to identify processes that are most relevant for prevention or treatment.

## 1. Introduction

Vascular calcification is a frequent feature of aging, atherosclerosis, diabetes and chronic kidney disease (CKD), but the term encompasses anatomically distinct lesions. Intimal calcification develops within atherosclerotic plaques and, in human coronary arteries, calcium burden closely reflects overall plaque burden [1]. Its clinical relevance is well established: coronary calcium strongly predicts future coronary events beyond conventional risk factors [2]. Medial arterial calcification, by contrast, develops predominantly along the elastic lamellae of the arterial media and can occur without an obstructive atherosclerotic plaque. It is particularly prominent in diabetes and advanced CKD, where it is associated with arterial stiffness, cardiovascular events and mortality [3,4]. Intimal and medial mineralization may nevertheless coexist, particularly in advanced systemic disease.

This anatomical distinction is biologically important. Intimal mineralization evolves within the lipid-rich, inflammatory environment of an atherosclerotic lesion, whereas medial calcification primarily involves smooth muscle cells, elastic lamellae and the extracellular matrix of the arterial wall [5]. Their initiating conditions and consequences therefore need not be identical, even when the mineral ultimately deposited is similar.

Against this heterogeneous background, no single mechanism adequately explains vascular calcification across disease settings. Here, we examine 14 experimentally supported mechanisms and organize them into five functional levels. The hierarchy is intended as a framework for causal organization rather than a temporal sequence: different diseases may enter the process at different points and engage the same mechanisms to different degrees. This perspective allows vascular calcification to be considered as a family of related pathologies while preserving the important distinction between where mineral develops and how the surrounding vascular tissue responds. As a narrative mechanistic review, the literature was selected to emphasize primary studies providing functional or causal evidence, including genetic, pharmacological, ex vivo and human studies. The 14 mechanisms were chosen as experimentally supported and functionally distinct processes rather than as an exhaustive catalog of all molecular regulators associated with vascular calcification. Table S1 summarizes representative studies that introduced or substantially advanced these mechanistic concepts. Because the relative contribution of these mechanisms varies with anatomical and disease context, the predominant vascular compartment and representative experimental settings discussed throughout the review are summarized in Table S2.

## 2. Historical Perspective

The conceptual history of vascular calcification reflects a progressive expansion in how the disease has been understood (Figure 1). The importance of active mineral control was recognized before the emergence of the modern vascular calcification field, when pyrophosphate was identified as an inhibitor of calcification [6]. The subsequent identification of matrix vesicles in calcified arteries provided a structural basis for local mineral nucleation [7]. Detection of BMP2 in human atherosclerotic lesions and the striking arterial phenotype of *Mgp*-null mice then established that vascular mineralization is subject to biological regulation rather than being determined by mineral chemistry alone [8,9]. The demonstration of osteochondrogenic proteins in human arteries subsequently placed VSMC plasticity at the center of this emerging model [10].

The early 2000s broadened the field substantially. Phosphate, inflammation and apoptosis were independently shown to promote vascular calcification [11,12,13], while fetuin-A–mineral complexes introduced the concept that nascent calcium phosphate can be packaged and stabilized rather than simply deposited in tissue [14]. Human *ENPP1* deficiency and experimental studies of extracellular pyrophosphate metabolism further established mineral inhibition as a physiological requirement for maintaining a non-calcified arterial wall [15,16].

Later work expanded this framework to include the mechanisms that determine how vascular cells tolerate and respond to persistent mineral stress. Oxidative stress, cellular senescence, endothelial plasticity, ER stress and autophagy showed that calcification-prone vascular tissue cannot be understood through osteogenic conversion alone [17,18,19,20]. Particularly informative was the demonstration that mitochondrial dysfunction in progeria reduces ATP availability and extracellular pyrophosphate, directly linking cellular bioenergetic failure to loss of extracellular mineral inhibition [21]. Genetic deletion of RUNX2 in VSMCs subsequently established a causal contribution of osteogenic reprogramming to lesion expansion [22], while more recent studies have extended regulated cell-death mechanisms beyond apoptosis to include ferroptosis.

Viewed chronologically, these observations progressively addressed different aspects of the same problem: whether calcium phosphate can form, where it nucleates and persists, how vascular cells respond to it, how those responses become self-sustaining, and what happens when cellular homeostasis can no longer contain mineral stress. The historical progression summarized in Table S1 therefore provides the conceptual basis for the five functional levels proposed here.

## 3. A Hierarchical Framework for Vascular Calcification

The mechanisms underlying vascular calcification do not occupy equivalent causal positions. We assigned each mechanism according to its predominant causal role: whether it primarily determines mineral permissiveness (Level I), mineral packaging, nucleation and retention (Level II), cellular reprogramming (Level III), amplification and persistence of the calcification-prone state (Level IV), or stress containment and homeostatic failure (Level V) (Table 1; Figure 2). Assignment to a level is therefore not exclusive. Mechanisms can influence other levels through feedback or secondary effects; for example, mitochondrial dysfunction can reduce ATP-dependent pyrophosphate production, extracellular vesicle release can increase during cellular stress, and inflammation can both precede and follow mineral formation. These major cross-level interactions, together with the principal supporting evidence and limitations for each mechanism, are summarized in Table S3. The choice of five levels is pragmatic rather than absolute: it preserves five distinct functional questions without implying a fixed temporal sequence or preventing cross-level interactions. The framework extends our previous emphasis on mineral permissiveness and active inhibition by incorporating mineral handling, cellular reprogramming, amplification and homeostatic failure within the same causal architecture. Level I, mineral permissiveness, determines whether the extracellular environment can sustain a calcium-phosphate phase. Elevated calcium and phosphate increase mineral pressure, whereas pyrophosphate, matrix Gla protein, fetuin-A and related defenses oppose nucleation and crystal growth [23]. The importance of this level is demonstrated particularly clearly by genetic loss of MGP or ENPP1, which is sufficient to produce severe arterial mineralization [9,15]. Likewise, both devitalized aortas ex vivo [24] and VSMCs in vitro [25] can mineralize under permissive calcium-phosphate conditions, showing that neither transcription nor osteogenic differentiation is required for mineral formation itself [24,25,26].

Level II, mineral packaging, nucleation and lesion growth, determines what happens once calcium phosphate begins to leave the ionic state. Fetuin-A captures nascent mineral within calciprotein particles, initially limiting uncontrolled precipitation [14]. Cell-derived extracellular and matrix vesicles provide phospholipid-rich surfaces on which calcium phosphate can nucleate, while extracellular matrix architecture determines whether these nanoscale structures are retained and incorporated into a growing lesion [27]. This level therefore converts mineral pressure into spatially organized tissue mineralization.

Level III, cellular reprogramming, describes how vascular cells respond to and reshape this altered environment. VSMCs can enter osteochondrogenic states, and lineage-specific RUNX2 deletion reduces arterial calcification, demonstrating that cellular plasticity contributes directly to lesion expansion [22,28]. BMP–WNT signaling and endothelial-to-mesenchymal transition broaden this response by stabilizing osteogenic programs or increasing the pool of cells capable of entering mesenchymal and osteogenic states. These pathways can precede detectable mineral in inflammatory disease, but they need not generate the first mineral nucleus.

Level IV, amplification and memory, comprises inflammation, oxidative and mitochondrial dysfunction, senescence, and epigenetic–metabolic reprogramming. These mechanisms amplify calcification-prone responses and can stabilize altered cellular states after the initiating stimulus has weakened. This level provides the system with biological hysteresis: the vessel becomes progressively less able to return to its original mineral-resistant state.

Finally, Level V, stress containment and homeostatic failure, determines whether damaged vascular cells recover or become additional sources of mineralizing material. Adaptive stress responses initially preserve cellular homeostasis, whereas their failure promotes vesicular release, regulated cell death and the generation of new mineralization substrates.

The five levels should therefore not be interpreted as five stages of vascular calcification. They define predominant causal roles within a coupled system, and different disease settings may enter this system through different levels and engage them to different degrees (Figure 2).

This distinction also requires separating physicochemical sufficiency, biological necessity and temporal priority. Demonstrating calcium-phosphate deposition in fixed cells or devitalized arteries shows that active transcription is not necessary for mineral formation under permissive conditions, but it does not establish that mineral is invariably the first event in vivo. Conversely, inflammation, matrix remodeling or phenotypic reprogramming may precede detectable mineral in particular disease settings without necessarily being sufficient to generate mineral in the absence of a permissive extracellular environment. The causal order should therefore be considered disease- and model-dependent rather than as a universal mineral-first sequence.

### 3.1. Level I. Mineral Permissiveness

#### 3.1.1. Elevated Phosphate and Calcium: Supersaturation and Mineral Nucleation

Elevated extracellular phosphate is one of the most reproducible drivers of vascular calcification, particularly in chronic kidney disease (CKD) [29,30]. Its mechanism, however, has been interpreted in two rather different ways. The influential cellular model proposed that inorganic phosphate (Pi) enters vascular smooth muscle cells (VSMCs) through type III sodium-dependent phosphate transporters, particularly PiT-1, and activates an osteogenic program culminating in RUNX2 expression and mineral deposition [31,32]. A substantial body of experimental work now indicates that this sequence is incomplete [33]. Phosphate is not only a cellular signal or metabolic substrate; together with calcium, it is a structural component of the mineral itself [23,25,34]. Increasing either ion changes the physicochemical state of the extracellular compartment and increases the probability that calcium phosphate will leave solution [35]. The critical event may therefore occur outside the cell, before osteogenic reprogramming begins.

The transporter model originated from experiments in human aortic smooth muscle cells in which increasing Pi above physiological concentrations caused a dose-dependent increase in mineral deposition accompanied by induction of CBFA1/RUNX2 and osteocalcin [11,36]. Stable depletion of PiT-1 markedly reduced phosphate uptake, osteogenic gene induction and mineralization, whereas re-expression of PiT-1 restored the phenotype [31]. In addition, phosphonoformic acid (PFA) inhibited both responses (phosphate transport and phosphate-calcium deposition), but was interpreted as blocking sodium-dependent phosphate transport [37]. Subsequent genetic experiments appeared to strengthen this conclusion. These studies established an important point that remains valid: PiT-1 participates in the response of VSMCs to a high-phosphate environment. What they did not establish definitively was that increased phosphate flux through PiT-1 is the event that initiates mineralization.

Direct measurements of phosphate transport exposed the first difficulty with that interpretation. PiT-1 and PiT-2 are high-affinity transport systems, and sodium-dependent as well as sodium-independent phosphate uptake in primary VSMCs is already close to saturation at physiological extracellular Pi concentrations [33]. Calcification, by contrast, rises sharply when extracellular phosphate is increased into the millimolar range. The concentration dependence of mineralization therefore does not mirror the concentration dependence of phosphate uptake. Moreover, phosphate, PDGF-BB and TNF-α can all increase calcification and osteogenic gene expression without a corresponding increase in phosphate transport across the plasma membrane. PiT-1 itself is substantially intracellular under basal conditions and can accumulate within the endoplasmic reticulum rather than at the cell surface [38].

The interpretation of PFA proved even more consequential. Concentrations of PFA that completely suppress calcification have little effect on PiT-mediated phosphate uptake in VSMCs. More strikingly, PFA prevents calcium-phosphate deposition in paraformaldehyde-fixed cells, in which membrane transport, signaling and transcription are absent [37]. Pyrophosphate and related phosphonates show similar behavior. PFA therefore acts largely by interfering directly with calcium-phosphate deposition or crystal growth rather than by preventing cellular phosphate entry. This removes one of the original pharmacological arguments for an obligatory transport-dependent mechanism, without negating the genetic evidence that PiT proteins influence VSMC phenotype.

Once calcium-phosphate chemistry is considered explicitly, the steep dependence of calcification on extracellular phosphate becomes less surprising. Extracellular fluids contain calcium and phosphate at concentrations at which formation of calcium-phosphate phases is thermodynamically possible. Soft tissues remain unmineralized because nucleation and crystal growth are kinetically constrained and because extracellular inhibitors suppress the transition from soluble ions to a stable mineral phase. Raising phosphate increases supersaturation and lowers the barrier to formation of a persistent calcium-phosphate nucleus. Calcium is equally important in this process. Experiments using fixed VSMCs showed that mineral formation depends on the individual concentrations of Ca^2+^, Pi and hydroxyl ions rather than on a simple calcium × phosphate product. In these experiments with fixed VSMCs, an incubation medium containing 4 mmol/L phosphate and 1 mmol/L calcium failed to produce mineralization, whereas other experimental combinations with sufficient calcium produced extensive deposition [25]. Thus, phosphate cannot be considered in isolation from the calcium concentration with which it interacts.

Human vascular tissue provides a complementary perspective. In arterial rings from patients with CKD, prolonged exposure to elevated calcium or phosphate increased mineral deposition, and calcium was particularly potent when the two ions were compared at an equivalent calcium-phosphate product [39]. Healthy arterial rings were considerably more resistant to the same challenge. These findings indicate that the response depends not only on extracellular calcium and phosphate concentrations, but also on the capacity of the vascular wall to resist mineral deposition [39].

Perhaps the most direct demonstration of physicochemical sufficiency came from experiments separating mineral deposition from cellular metabolism. Both devitalized aortas and VSMCs mineralized readily in calcium- and phosphate-containing medium and in some conditions accumulated mineral faster than viable aortas or cells [24,25]. These experiments establish that calcium-phosphate deposition can occur without active cellular transcription under permissive experimental conditions; they do not establish its temporal priority in human vascular disease.

Hydroxyapatite was the final crystalline phase. More importantly, 4 mmol/L extracellular phosphate did not induce BMP2 or CBFA1/RUNX2 when calcium was kept sufficiently low to prevent calcium-phosphate deposition. The same phosphate concentration induced these genes when mineral deposition was permitted. In this experimental setting, soluble phosphate alone was therefore insufficient to trigger the classical osteogenic response. The appearance of a calcium-phosphate phase coincided with, and could precede, induction of an osteogenic response characterized by increased BMP2 and CBFA1/RUNX2 expression [24,25].

Independent experiments reached a remarkably similar conclusion by examining what actually forms in phosphate-enriched culture medium. Increasing phosphate by only 1–2 mmol/L generated nanoscale calcium-phosphate particles within approximately one day. Their formation required both calcium and phosphate and was strongly inhibited by fetuin-A. Purified calcium-phosphate nanocrystals induced BMP2 and osteopontin in smooth muscle cells, whereas high-phosphate medium from which the particles had been removed no longer produced the same response [26]. Part of what has historically been described as “phosphate signaling” can therefore be mediated by a new physical entity generated by phosphate: a calcium-phosphate nanoparticle. Once ions enter a particulate mineral phase, they acquire biological properties that soluble phosphate does not possess.

These observations change, rather than eliminate, the role of the phosphate transporters. Later experiments from the Giachelli laboratory independently confirmed that maximal phosphate uptake occurs at concentrations considerably below those required for osteochondrogenic differentiation and mineralization [40]. PiT-1 deficiency impaired phosphate-induced ERK1/2 activation, but a transport-deficient PiT-1 mutant restored ERK signaling and substantially rescued the osteochondrogenic phenotype. Mineralization was restored more completely by transport-competent PiT-1, indicating that phosphate transport still contributes to the response [40]. However, the rescue produced by the transport-deficient mutant shows that PiT-1 also has functions independent of phosphate translocation. These findings indicate that PiT-1 is not simply a conduit for phosphate entry, but also participates in signaling pathways that influence the osteochondrogenic response. The in vivo data reinforce this more nuanced interpretation. Smooth-muscle-specific deletion of PiT-1 did not reduce medial calcification in mice with CKD subjected to phosphate loading. PiT-2 increased in the PiT-1-deficient media and compensated for phosphate uptake, leading initially to the conclusion that the two proteins have redundant functions [32]. Partial loss of PiT-2 produced the opposite phenotype. Sodium-dependent phosphate uptake was reduced, yet vascular calcification increased in uremic mice [41]. PiT-2-deficient VSMCs also showed lower osteoprotegerin expression and greater susceptibility to mineralization. These findings show that reduced phosphate transport can coexist with increased calcification. The biological effects of SLC20 transporters therefore cannot be inferred solely from the magnitude of cellular Pi uptake. These findings support a model in which extracellular phosphate acts at two mechanistically distinct levels. At the most fundamental level, Pi and Ca^2+^ increase supersaturation and the probability that a persistent calcium-phosphate phase will form, a process that requires neither phosphate transport nor an osteogenic transcriptional program. Living vascular cells then respond to this altered mineral environment, with PiT proteins and other signaling pathways modifying the cellular phenotype. RUNX2 remains an important determinant of VSMC osteogenic reprogramming, but its induction after phosphate exposure does not establish that soluble phosphate directly initiates osteogenic differentiation; calcium-phosphate deposition can precede RUNX2 induction, and nanocrystals themselves can trigger osteogenic signaling [24,25,26]. For phosphate-driven experimental calcification, a useful starting model is therefore not phosphate → PiT-1 → RUNX2 → hydroxyapatite, but mineral pressure → nucleation → cellular response and amplification. PiT-1 and PiT-2 modify this response but cannot be reduced to a simple relationship between phosphate uptake and calcium deposition.

#### 3.1.2. Failure of Mineralization Inhibitors

If elevated calcium and phosphate increase the physicochemical tendency for calcium-phosphate mineral to form, the persistence of a non-calcified arterial wall requires active inhibition. Vascular extracellular fluids are metastable: calcium-phosphate precipitation is possible, but normally restrained by soluble and matrix-associated defenses. Calcification can therefore arise not only from increasing mineral load, but when this inhibitory capacity becomes inadequate.

Inorganic pyrophosphate provides the clearest demonstration. Intact rat aortas remain resistant to mineralization despite exposure to high calcium and phosphate, whereas enzymatic removal of pyrophosphate triggers hydroxyapatite deposition and exogenous pyrophosphate prevents it [21,42,43,44]. Vascular cells generate pyrophosphate from extracellular ATP through ENPP1, while tissue-nonspecific alkaline phosphatase (TNAP) hydrolyzes pyrophosphate to phosphate; manipulating either reaction correspondingly changes arterial calcification [24,45]. Human genetics establishes the importance of this balance particularly strongly: loss-of-function mutations in *ENPP1* cause generalized arterial calcification of infancy [15]. These genetic disorders provide mechanistic evidence that disruption of the pathway is sufficient to promote arterial mineralization, but they should not be regarded as direct models of common acquired vascular calcification.

Pyrophosphate protection is both local and systemic, and the same pathway can be impaired in acquired disease without a germline mutation. Vascular transplantation experiments showed that calcification follows the circulating pyrophosphate environment rather than the arterial genotype alone [46], while hepatic ABCC6-dependent ATP release provides a major source of systemic pyrophosphate [47,48]. CKD further weakens this defense through reduced circulating pyrophosphate and increased vascular TNAP-mediated hydrolysis, whereas pyrophosphate administration suppresses uremic vascular calcification [42,49,50].

Importantly, failure of mineral inhibition does not necessarily require reduced expression or activity of an inhibitor. During phosphate-induced calcification, VSMCs can actually increase ENPP1 activity and pyrophosphate synthesis, apparently as a compensatory response [24]. However, this increase may remain insufficient relative to the prevailing calcium-phosphate load. Mineralization can therefore progress despite activation of an endogenous inhibitory response.

Matrix Gla protein (MGP) provides a complementary local barrier. *Mgp*-null mice develop rapid and catastrophic arterial calcification, demonstrating that an intact vascular matrix must actively resist mineral deposition [9]. MGP function requires vitamin K-dependent γ-carboxylation; warfarin therefore creates functional deficiency despite continued protein expression [51]. This distinction is relevant because MGP can accumulate in calcified vessels without providing adequate protection. MGP-mediated protection is also influenced by the status of other mineralization inhibitors. In uremic arteries, loss of functional MGP becomes more consequential when pyrophosphate protection is simultaneously reduced [52].

Fetuin-A acts differently. Rather than acting principally as a local matrix-associated inhibitor, circulating fetuin-A binds nascent calcium-phosphate clusters and stabilizes them as soluble mineral–protein colloids [14]. Its physiological importance is evident from the severe susceptibility of fetuin-A-deficient mice to ectopic calcification during mineral loading or CKD [53,54,55]. Fetuin-A can also enter VSMC-derived vesicles, where it reduces their capacity to nucleate calcium phosphate [56]. Thus, systemic mineral buffering and local nucleation control are closely connected.

Osteopontin illustrates a different form of protection. It is induced around established mineral rather than simply disappearing before calcification. In Mgp-deficient mice, genetic deletion of osteopontin markedly accelerates arterial mineralization, supporting a protective role for osteopontin in limiting crystal growth and lesion propagation in this sensitized genetic background [57].

The vascular anti-mineralization system is therefore layered and partly redundant. Pyrophosphate restrains crystal formation and growth, MGP protects the extracellular matrix, fetuin-A captures nascent mineral in the circulation, and osteopontin limits expansion of established deposits. Failure may result from deficient synthesis, accelerated degradation, defective post-translational activation or simply mineral pressure sufficient to overwhelm otherwise intact defenses. The transition from this permissive mineral environment to nucleation, mineral packaging and organized vascular mineralization is summarized in Figure 3.

### 3.2. Level II. Mineral Packaging, Nucleation and Lesion Growth

#### 3.2.1. Calciprotein Particles

Fetuin-A does not prevent calcium phosphate from entering a solid phase; it changes the form in which that mineral appears. In supersaturated solutions, fetuin-A rapidly binds nascent calcium-phosphate clusters and stabilizes them as soluble calciprotein particles (CPPs) rather than allowing uncontrolled precipitation [14]. CPP formation should therefore be viewed initially as a protective mineral-buffering response. The earliest primary CPPs (CPP-I) are small, relatively soluble particles containing predominantly amorphous calcium phosphate. With time, they undergo structural maturation into larger, less soluble secondary CPPs (CPP-II) containing increasingly crystalline mineral [58,59]. This transition changes not only particle morphology, but also surface charge, solubility, protein composition and biological behavior.

The physiological purpose of mineral packaging is also evident from clearance studies. Fetuin-A-containing CPPs are rapidly removed by the reticuloendothelial system, whereas maturation into more crystalline secondary CPPs alters their cellular handling and prolongs their persistence [60,61]. CPP maturation therefore creates a biologically distinct particle rather than simply a larger version of the same one.

This distinction also explains the apparent paradox that CPP formation can be protective while CPPs are associated with vascular injury. Fetuin-A-containing particles are substantially less cytotoxic than equivalent amounts of naked hydroxyapatite, indicating that mineral packaging reduces the immediate toxicity of calcium phosphate [62]. The pathological problem arises when mineral generation exceeds stabilization and clearance. Persistent CPPs mature, acquire greater crystallinity and increasingly interact with vascular and immune cells.

This transition is particularly relevant to VSMCs. Primary CPPs cause little mineralization, whereas secondary CPPs induce pronounced VSMC calcification, oxidative stress and endogenous TNF-α production; silencing TNF-α or TNFR1 attenuates this effect [63]. Magnesium provides an unusually clean physicochemical test of the mechanism. Increasing Mg^2+^ delays CPP-I-to-CPP-II maturation and prevents phosphate-induced VSMC calcification, but loses much of this protective effect when preformed CPP-IIs are applied directly to the cells [64]. Part of what appears experimentally as “phosphate toxicity” can therefore occur upstream of the VSMC, through phosphate-dependent generation and maturation of the mineral particles to which the cell is subsequently exposed.

Crystallinity should not be interpreted as a simple progression from harmless CPP-I to toxic CPP-II. Both particle states are biologically active but elicit different responses: primary CPPs can favor IL-1β-associated inflammatory stress, whereas secondary particles preferentially stimulate TNF-α and strongly promote VSMC calcification [61,63,65]. Maturation therefore changes the type of biological stress rather than simply increasing toxicity.

Particles isolated from advanced CKD further support the importance of composition as well as crystallinity. CPP preparations from patients with stage 5 CKD contained less fetuin-A and Gla-rich protein and showed greater mineral maturation than those from healthy individuals; these particles promoted VSMC inflammatory, osteochondrogenic and calcifying responses, whereas γ-carboxylated GRP reduced their activity [66]. CPPs can also impair endothelial nitric oxide signaling and endothelium-dependent relaxation [67], while experimental evidence suggests that mineral-containing particles may participate in physiological phosphate sensing and FGF23 regulation in bone [68]. CPPs are therefore not intrinsically pathological structures. Their biological meaning depends on mineral load, maturation state, the composition of the protein corona, that is, the layer of proteins associated with the particle surface, and on clearance and target cell. Human CPP measurements should nevertheless be interpreted cautiously because sample handling can alter particle size and crystallinity, while the serum T50 assay measures calcification propensity rather than circulating CPP concentration [69,70].

CPPs thus occupy the transition between successful mineral defense and pathological mineral stress. Fetuin-A converts nascent calcium phosphate into a transportable colloid; efficient clearance prevents that material from precipitating into tissue. When mineral generation persists, however, particle maturation changes how the mineral is recognized, internalized and processed by vascular and immune cells. The result is a route by which an extracellular chemical disturbance becomes cellular oxidative, inflammatory and procalcific stress.

#### 3.2.2. Extracellular and Matrix Vesicles

Calciprotein particles begin with mineral captured by soluble proteins; vascular extracellular vesicles begin with a cell-derived membrane. This distinction is important because it places extracellular vesicles (EVs) at a different point in mineral formation. Rather than transporting mineral that has already emerged from solution, calcification-competent EVs provide a restricted membrane surface on which calcium and phosphate can concentrate and nucleate locally. They therefore offer a physical mechanism by which cellular stress is converted into extracellular mineral.

Matrix-vesicle-like structures were identified by electron microscopy in calcifying human arteries several decades ago [7], but their functional importance became clearer in cultured human VSMCs. Increasing extracellular calcium or phosphate generated both apoptotic bodies from dying cells and matrix vesicles from viable cells, and both could nucleate basic calcium phosphate [27]. Importantly, vesicle release itself was not sufficient for calcification. Vesicles generated in the presence of serum incorporated fetuin-A and MGP and were much less mineralization competent. The critical variable was therefore vesicle composition rather than vesicle abundance alone.

This distinction is particularly evident in CKD. Arteries from dialysis patients exposed ex vivo to elevated calcium and phosphate release abundant VSMC-derived vesicles containing crystalline calcium phosphate, whereas normal vessels remain considerably more resistant under the same conditions [39]. Mineral-containing vesicles can appear adjacent to morphologically intact VSMCs, suggesting that vesicular export may initially represent an adaptation to disturbed calcium homeostasis. What protects the individual cell by moving calcium away from the cytosol, however, transfers the mineral burden to the extracellular matrix.

The molecular surface of the vesicle determines whether this adaptation becomes pathological. Vesicles from calcifying VSMCs are enriched in annexins, alkaline phosphatase and calcium and can mineralize type I collagen independently of the cells that produced them [71]. In primary human VSMCs, extracellular calcium promotes translocation of annexin A6 to the membrane, exposure of phosphatidylserine and release of vesicles enriched in annexins A2, A5 and A6 but relatively depleted of MGP [72]. Annexin A6 depletion reduces mineralization. Negatively charged phosphatidylserine provides a calcium-binding surface, while annexins stabilize calcium-rich membrane domains, creating a plausible site for the first persistent calcium-phosphate clusters.

Vascular matrix-vesicle formation resembles physiological skeletal mineralization, but in arteries these structures can arise as a stress response of VSMCs rather than as evidence of complete osteoblastic conversion.

The intracellular origin of at least one major EV population was established by tracing vesicle trafficking using fluorescently labeled fetuin-A. In human VSMCs, Alexa488-labeled fetuin-A was internalized into endosomal compartments, colocalized with markers of multivesicular bodies, and was subsequently released through the exosomal pathway [73]. Their secretion depends on SMPD3/nSMase2, and elevated extracellular calcium increases both SMPD3 expression and vesicle release; pharmacological inhibition of SMPD3 reduces calcification. Multivesicular bodies and CD63-positive structures were also identified in calcifying arteries from dialysis patients. Thus, a substantial fraction of vascular matrix vesicles has an exosomal origin, although the heterogeneous EV populations recovered experimentally should not all be assumed to share identical biogenesis.

Their mineralizing potential is determined as much by cargo as by membrane composition. Sortilin provides particularly strong evidence. In calcifying VSMCs, sortilin is recruited into EVs and controls vesicular loading of tissue-nonspecific alkaline phosphatase (TNAP), thereby concentrating an enzyme that simultaneously destroys pyrophosphate and generates phosphate at the nucleation site [74]. *Sort1* deficiency reduces vascular calcification in vivo without impairing skeletal mineralization. The experiment links vesicle trafficking directly to the extracellular pyrophosphate chemistry discussed earlier: a membrane rich in phosphatidylserine and annexins can concentrate calcium while locally delivered TNAP removes one of the principal restraints on crystal growth.

Conversely, EVs can carry mineralization inhibitors. Fetuin-A, MGP and γ-carboxylated proteins such as prothrombin can restrain calcium-phosphate nucleation on phosphatidylserine-rich vesicle surfaces, linking vesicle composition to vitamin K-dependent mineral protection [56,75].

EVs are also capable of transmitting a calcification-prone state between cells. Matrix vesicles derived from CKD VSMCs are internalized by otherwise noncalcifying VSMCs and increase intracellular calcium, oxidative signaling and subsequent mineralization through pathways including MEK–ERK and NOX1 [76]. ER stress similarly changes both the amount and composition of released vesicles: stressed human VSMCs increase SMPD3-dependent secretion of GRP78-enriched EVs, and reducing GRP78 attenuates calcification [77]. EVs are therefore not merely passive seeds. Their cargo can communicate the metabolic and proteostatic state of the donor cell to its neighbors.

The transition from nanometer-scale vesicles to macroscopic vascular calcification requires one further element: retention within extracellular matrix. In three-dimensional collagen systems and human atherosclerotic plaques, calcifying EVs become trapped between collagen fibers, aggregate and mineralize, producing spherical microcalcifications that subsequently coalesce into larger deposits [78]. Collagen density determines how freely vesicles cluster and therefore helps determine mineral geometry. Sequential mineral labeling in vivo confirmed that pre-existing microcalcifications are incorporated into larger calcified structures. This work provides a physical bridge between a membrane vesicle released by a single VSMC and a lesion visible at the tissue level.

Extracellular vesicles therefore link cellular stress to extracellular mineral nucleation. Their mineralizing capacity depends on membrane composition, the local phosphate/pyrophosphate balance, inhibitory proteins and disease-associated changes in vesicle cargo.

Once released, however, EVs cease to be governed only by the cell that produced them. Whether they remain dispersed, are cleared, or develop into persistent microcalcifications depends increasingly on the extracellular scaffold into which they are deposited. Elastin, collagen architecture, proteolytic remodeling and matrix stiffness determine where vesicles are retained and how their mineral phase expands. The next step in the process is therefore not another vesicle pathway, but the extracellular matrix that converts dispersed nanoscale mineralization events into an organized vascular lesion.

#### 3.2.3. Extracellular Matrix Remodeling and Degradation

The extracellular matrix is not merely the substrate on which vascular mineral eventually accumulates. Its composition, architecture and mechanical properties determine whether calcium-phosphate nuclei persist and how vascular smooth muscle cells (VSMCs) respond to them. Early experiments already showed that type I collagen and fibronectin promote vascular-cell mineralization, whereas type IV collagen is inhibitory; transferring cells onto a calcification-prone matrix was sufficient to accelerate mineral deposition [79]. Calcification susceptibility therefore resides partly in the extracellular environment rather than exclusively in the intrinsic state of the cell.

Elastin is particularly important because medial calcification frequently develops along elastic lamellae. Proteolytic fragmentation exposes new mineral-binding surfaces and releases biologically active elastin peptides. Elastin-derived peptides can induce RUNX2, alkaline phosphatase and osteocalcin in VSMCs, particularly in the presence of TGF-β1 [80], whereas elastin fragments markedly amplify phosphate-induced mineralization without producing substantial calcification at normal phosphate concentrations [81]. Matrix damage is therefore permissive rather than universally sufficient.

The strongest causal evidence comes from manipulating elastolysis in vivo. In ApoE-deficient mice with CKD, deletion of the potent elastase cathepsin S preserved elastic lamellae and markedly reduced arterial calcification despite comparable renal dysfunction and macrophage accumulation [82]. MMP-2 and MMP-9 similarly increase during experimental CKD calcification, and broad MMP inhibition reduces mineralization of arterial tissue [83]. Protease activity thus does more than accompany calcification: it changes the material environment in which mineral can form.

Temporal experiments nevertheless show that elastin degradation alone is not enough. In uremic mice, elastin turnover and loss of the differentiated VSMC phenotype precede detectable medial calcification, but substantial mineralization occurs only when the animals are also exposed to a sufficiently high phosphate burden [84]. This is a useful constraint on the mechanism. Matrix injury lowers the threshold for calcification; it does not abolish the requirement for a permissive calcium-phosphate environment.

The *Mgp*-deficient model provides an even more direct challenge to a strictly cell-first sequence. Mineral initially appears along elastic lamellae before detectable induction of major osteochondrogenic markers. Reducing elastin genetically in *Mgp*−/− mice significantly reduces arterial calcification [85]. Here, the amount of available elastin helps determine the extent of mineral deposition, and extracellular mineralization can precede overt osteogenic reprogramming.

Once mineral is present, the direction of signaling reverses. VSMCs cultured on hydroxyapatite or calcified elastin lose contractile markers and increase RUNX2, alkaline phosphatase and osteochondrogenic genes; removal from the calcified environment partially restores their original phenotype [86]. Marfan syndrome provides a translational example of how a primary matrix disorder can promote vascular mineralization. In human Marfan aortas, medial microcalcification is associated with extensive elastic lamina fragmentation, and a similar relationship is observed in Fbn1^C1039G/+ mice [87]. Mechanistically, elastin-derived peptides increase alkaline phosphatase activity and reduce MGP expression in VSMCs through signaling involving the elastin receptor complex and ERK1/2 [87]. These findings link a genetically determined matrix defect to elastin injury, altered VSMC signaling and local microcalcification across human and experimental systems. Together, these observations show that mineralized or degraded matrix can actively influence VSMC phenotype rather than remaining an inert consequence of disease. Collagen remodeling adds a mechanical dimension. Increased LOX- and PLOD1-dependent collagen cross-linking promotes VSMC mineralization, whereas inhibition of either enzyme reduces it [87]. More directly, increasing substrate stiffness enhances osteogenic differentiation through loss of nuclear monomeric actin and activation of YAP–TEAD and YAP–RUNX2 signaling; experimentally stiffening human vascular tissue produces the same effect [88]. Thus, fibrosis and calcification can reinforce one another through mechanotransduction even without changing extracellular phosphate.

Matrix architecture also determines how nanoscale mineral becomes a macroscopic lesion. Calcifying extracellular vesicles are retained between collagen fibers, aggregate and fuse into microcalcifications that subsequently become incorporated into larger deposits [78]. Collagen density influences the size and spatial organization of these aggregates. The matrix therefore controls not only whether mineral nucleates, but how independent nucleation events are assembled into the geometry of a calcified plaque.

In this conceptual sense, extracellular matrix remodeling can act as a form of structural memory. Elastolysis exposes mineral-permissive surfaces and releases signaling matrikines, collagen deposition and cross-linking alter stiffness and mechanotransduction, and matrix architecture determines vesicle retention and mineral growth. These persistent structural changes can maintain a calcification-prone local environment even after the initiating systemic disturbance has diminished, thereby facilitating further mineral deposition and VSMC phenotypic change.

### 3.3. Level III. Cellular Reprogramming

#### 3.3.1. Osteogenic Reprogramming and VSMC Plasticity

Once mineral has begun to accumulate within a remodeled vascular matrix, the surrounding cells do not remain phenotypically neutral. Vascular smooth muscle cells (VSMCs) can relinquish elements of their contractile identity and acquire transcriptional programs normally associated with osteochondrogenic development. This response is now supported by human pathology, genetic fate mapping and lineage-specific manipulation, although the term *transdifferentiation* probably implies a cleaner conversion than actually occurs.

Early human studies provided the first indication that calcified arteries undergo an organized change in cell phenotype. Medial calcification in Mönckeberg sclerosis was associated with expression of alkaline phosphatase, bone sialoprotein, osteocalcin and type II collagen in regions containing VSMCs, and cells isolated from these lesions retained an enhanced capacity to mineralize in vitro [10]. Calcified human atherosclerotic lesions similarly contained vascular cells expressing RUNX2, MSX2 and SOX9 together with osteogenic and chondrogenic matrix proteins [89]. In arteries from patients with kidney failure, RUNX2 and osteopontin were enriched in calcified regions [90]. Their presence in the same regions does not imply equivalent functions: RUNX2 is consistent with osteogenic reprogramming, whereas osteopontin can be induced around established mineral as a counter-regulatory response that limits crystal growth [57,91]. These observations established an association between mineral deposition and altered vascular-cell phenotype, but could not determine where the osteogenic cells had originated.

Experimental models showed that this phenotype remains remarkably plastic. Calcifying conditions reduce smooth-muscle markers while increasing RUNX2, alkaline phosphatase, osteocalcin and osteopontin [11,91]. More importantly, VSMCs isolated from calcifying *Mgp*-deficient arteries can recover smooth-muscle markers when returned to a differentiation-promoting environment and subsequently re-enter an osteochondrogenic state when challenged again. Manipulating RUNX2 showed that its induction, rather than loss of myocardin alone, is required for much of this lineage reprogramming [92]. Loss of contractile identity and acquisition of osteogenic identity are therefore related but not equivalent events.

Genetic fate mapping provided stronger evidence of origin. In *Mgp*-deficient mice, permanently labeled smooth-muscle-lineage cells gave rise to osteochondrogenic precursor- and chondrocyte-like cells within calcifying arteries [28]. In atherosclerotic mice, approximately 80% of RUNX2-positive cells and almost all type II collagen-positive chondrocyte-like cells originated from the smooth-muscle lineage [93]. These experiments established VSMCs as a major cellular source of the osteochondrogenic population rather than merely cells located adjacent to mineral. Importantly, however, bone-marrow-derived cells also contributed, indicating from the outset that vascular osteogenesis is not lineage exclusive.

RUNX2 is functionally involved in this transition rather than serving only as a marker. Smooth-muscle-specific *Runx2* deletion reduces atherosclerotic intimal calcification in mice [94]. Using an improved conditional allele, Lin and colleagues subsequently showed that VSMC-specific *Runx2* deletion reduced arterial calcification by roughly half without significantly changing atherosclerotic lesion size, macrophage burden or systemic mineral parameters [22]. These findings support a causal contribution of RUNX2-dependent VSMC reprogramming to lesion expansion. They do not, however, imply that RUNX2 activation is the initiating event in all forms of vascular calcification.

The modern view is nevertheless broader than a direct VSMC-to-osteoblast conversion. Lineage tracing combined with single-cell transcriptomics shows that diseased VSMCs move through heterogeneous intermediate states in which canonical smooth-muscle genes may be profoundly suppressed before more specialized phenotypes emerge. In advanced atherosclerosis, smooth-muscle-derived cells populate LGALS3-positive, progenitor-like states capable of giving rise to several downstream phenotypes, including osteogenic populations regulated in part by KLF4 [95]. Similar populations are detectable in human carotid lesions. Osteogenic differentiation is therefore better understood as one branch of VSMC plasticity rather than an obligatory terminal fate.

VSMCs are not the only vascular-wall cell population capable of contributing to osteogenic progenitor populations. In mice combining CKD and atherosclerosis, fate-traced adventitial GLI1-positive mesenchymal cells migrate into the media and intima and generate a substantial population of osteoblast-like cells; genetic ablation of these cells markedly reduces vascular calcification [96]. The relative contribution of resident VSMCs and alternative progenitor populations is therefore likely to depend on vascular compartment and disease context.

The emerging picture is not one in which vascular cells autonomously decide to “become bone.” Mineral stress, matrix remodeling and cellular plasticity interact. VSMCs provide a major source of osteochondrogenic cells, and RUNX2 is a causal component of that response, but the transition occurs through heterogeneous and partly reversible states rather than through a single irreversible lineage switch. The important mechanistic question consequently shifts from whether osteogenic reprogramming occurs to which extracellular signals stabilize it. Among the most consistently implicated are developmental pathways normally used during skeletal formation—particularly BMP and WNT signaling—which provide the focus of the next section.

#### 3.3.2. BMP–WNT Signaling

Osteogenic reprogramming of vascular cells is stabilized by developmental pathways normally used during skeletal formation. Among these, BMP and WNT signaling form a particularly important network. Their role, however, is better understood as amplification of a calcification-prone state than as a universal initiating cascade.

BMP2 was first identified in calcified human atherosclerotic lesions more than three decades ago [8]. Subsequent work linked BMP activity directly to vascular mineral regulation. Matrix Gla protein (MGP) binds BMP2 and suppresses BMP receptor signaling, providing a molecular connection between extracellular mineral inhibition and osteogenic signaling [97]. Consistent with this interaction, pharmacological inhibition of BMP type I receptors or sequestration of BMP ligands reduces vascular calcification in hyperlipidemic mice [98], while the same interventions suppress the severe medial calcification of *Mgp*-deficient mice by approximately 80% [99]. BMP signaling therefore contributes causally even in Mgp-deficient arteries, where loss of a major extracellular mineralization inhibitor strongly favors mineral deposition [9,99]. One of its most important downstream effects is the recruitment of a paracrine WNT program. In diabetic LDLR-deficient mice, activation of the BMP2–MSX2 axis accompanies vascular mineralization. Increasing MSX2 expression enhances Wnt3a and Wnt7a, suppresses the WNT antagonist Dkk1 and activates β-catenin-dependent transcription in neighboring cells; DKK1 reverses much of this activity [100]. Thus, osteogenic signaling can propagate through the vessel wall without requiring every responding cell to express the initiating signal.

Inflammation can drive this network. In diabetic LDLR-deficient mice, TNF-α neutralization reduces vascular BMP2, MSX2, WNT ligand expression and aortic calcium despite persistent obesity, hyperglycemia and hyperlipidemia [101]. In human VSMCs, TNF-α induces MSX2 through NF-κB and promotes alkaline phosphatase and osteogenic differentiation; MSX2 silencing suppresses much of this response [102]. These experiments place inflammatory signaling upstream of developmental pathways rather than treating inflammation and osteogenesis as independent mechanisms.

Canonical WNT signaling then provides a direct route to RUNX2. Stabilization of β-catenin promotes RUNX2 expression and calcification, whereas WNT inhibition suppresses both. Functional TCF/LEF-binding sites within the *RUNX2* promoter demonstrate that β-catenin can regulate RUNX2 transcription directly [103]. BMP2 and WNT signaling also cooperate experimentally: BMP2 increases β-catenin, MSX2 and RUNX2, while β-catenin depletion markedly reduces BMP2-associated calcification [104].

Yet the pathway is not a rigid BMP2 → MSX2 → WNT → RUNX2 sequence. BMP2 enhances VSMC calcification far more effectively in a permissive mineral environment than under normal phosphate conditions [105]. In VSMCs isolated from insulin-resistant ob/ob mice, MSX2 silencing reduced BMP2-dependent osteogenic differentiation but did not prevent BMP2-associated calcification [106]. Moreover, mineral formation itself can precede BMP2 and RUNX2 induction under some phosphate-rich conditions [24,25]. These observations separate the osteogenic program from the physical formation of calcium phosphate. BMP–WNT signaling strongly influences how vascular cells respond to a calcifying environment, defined here as a local extracellular milieu in which calcium-phosphate nucleation or growth is favored by increased mineral pressure, reduced inhibitory capacity, or pre-existing mineral. However, BMP–WNT signaling need not generate the first mineral nucleus. The most useful interpretation is therefore one of developmental amplification. BMP signals generated by VSMCs, endothelial cells or neighboring mesenchymal populations destabilize the mature vascular phenotype; MSX2 reshapes the local WNT environment; β-catenin reinforces RUNX2 and osteochondrogenic gene expression; and the resulting cells further alter matrix, alkaline phosphatase activity and vesicle biology. In diabetes, inflammation or atherosclerosis this program may precede substantial mineral deposition; in severe mineral stress it may be recruited by mineral that has already begun to form.

This broader role of BMP signaling also points beyond the smooth-muscle lineage. Endothelial cells exposed to excessive BMP, inflammatory or metabolic stress can lose endothelial identity and acquire mesenchymal and osteogenic competence. The same developmental pathways that reinforce VSMC plasticity therefore provide a natural entry into the next mechanism: endothelial-to-mesenchymal transition and the contribution of endothelial-derived cells to vascular calcification.

#### 3.3.3. EndMT and Alternative Cellular Sources

VSMCs are a major source of osteochondrogenic cells in calcifying arteries, but they are not the only vascular lineage capable of acquiring such properties. Endothelial cells can lose endothelial identity and enter mesenchymal intermediate states through endothelial-to-mesenchymal transition (EndMT). The important distinction is that EndMT does not usually represent a direct endothelial-to-osteoblast conversion. It creates a population with greater phenotypic plasticity, from which only a subset proceeds toward an osteochondrogenic fate.

A mechanistic precedent came from constitutively active ALK2 signaling, which caused endothelial cells to lose VE-cadherin and CD31 and acquire mesenchymal and multipotent characteristics capable of subsequent osteogenic differentiation [107]. Although this model reflects heterotopic ossification rather than ordinary vascular calcification, it established that excessive BMP signaling can destabilize endothelial lineage identity before osteogenesis occurs.

Direct vascular evidence emerged in *Mgp*-deficient and diabetic *Ins2*^Akita/+^ mice. Endothelial-marker-positive cells acquired SOX2, NANOG and OCT3/4 before expressing RUNX2 and Osterix, suggesting that BMP dysregulation first generates a less lineage-restricted state and only subsequently favors osteogenic differentiation [19]. This interpretation is particularly important in *Mgp*-null arteries, where loss of MGP simultaneously removes extracellular mineral inhibition and alters BMP signaling. EndMT therefore need not initiate the first mineral deposit; it can amplify a lesion already rendered permissive to calcification.

Endothelial SOX2 provides stronger causal evidence. In diabetic mice, reducing SOX2 within the endothelial compartment suppresses EndMT-associated phenotypes and decreases aortic calcium accumulation [108]. SOX2 appears to act less as an osteogenic transcription factor than as a regulator of cellular plasticity, loosening endothelial identity and increasing responsiveness to subsequent differentiation signals. Serine proteases induced downstream of BMP signaling contribute to this transition and simultaneously disrupt extracellular barriers, linking endothelial plasticity to matrix remodeling and migration into the vessel wall [109].

Atherosclerosis demonstrates why EndMT should not be equated with osteogenesis. Inducible endothelial lineage tracing identifies abundant endothelial-derived fibroblast-like cells within plaques, where they contribute to altered collagen and matrix-remodeling programs [110]. Thus, EndMT generates several possible mesenchymal trajectories; osteogenic differentiation is only one of them. Disturbed flow and inflammation promote this broader transition by weakening endothelial identity—for example, loss of endothelial FGFR1 releases TGF-β/SMAD signaling and accelerates EndMT in atherosclerosis [111].

Inflammatory signaling can subsequently determine whether an EndMT cell becomes osteogenic. TNF-α and IL-1β induce EndMT in primary human aortic endothelial cells and reduce BMPR2, making the transitioned cells markedly more susceptible to BMP9-driven osteogenic differentiation and mineralization [112]. The effect is not explained by a simple increase in canonical BMP signaling; inflammatory remodeling of receptor context changes how the endothelial cell interprets the BMP signal.

Recent work further narrows the claim. EndMT induced by TGF-β2 and IL-1β produces predominantly intermediate, oligopotent endothelial-derived cells rather than conventional mesenchymal stem cells. Only approximately 19% acquired substantial osteogenic and chondrogenic potential, while endothelial-lineage-traced cells in atherosclerotic mice formed an EndMT cluster enriched in ossification and cartilage-development programs [113]. EndMT therefore primes osteochondrogenic competence rather than determining it.

A similar sequence has recently been described under CKD-like mineral stress, where endothelial mesenchymal changes precede overt osteogenic gene expression and mineralization after phosphate exposure [114]. As with VSMCs, however, high-phosphate experiments cannot distinguish completely between responses to soluble phosphate and responses to calcium-phosphate particles generated in the medium. The stronger conclusion is that mineral stress can recruit an already plastic endothelial compartment into the calcifying process.

Endothelial involvement may also matter without direct mineral production. Partial EndMT disrupts junctions, increases migration and protease activity, alters collagen remodeling and changes paracrine communication with neighboring VSMCs. Endothelial-derived cells can therefore influence calcification even when they never become mineralizing osteoblast-like cells. This distinction is likely especially important in atherosclerotic intimal disease; the quantitative contribution of endothelial descendants to CKD-associated medial calcification remains less certain.

These findings indicate that EndMT broadens, rather than replaces, the VSMC-centered model. The calcifying artery contains several cell populations with substantial phenotypic plasticity. Resident VSMCs provide a major source of osteochondrogenic cells, adventitial progenitors can contribute in specific settings, and endothelial cells can generate heterogeneous mesenchymal descendants, only a fraction of which acquire osteogenic potential [28,95,96,108,109,110,111,112,113,114]. The fate of these populations is strongly influenced by the surrounding tissue environment. Among those environmental signals, inflammation is especially important. TNF-α and IL-1β can destabilize endothelial identity, alter BMP receptor signaling and influence the fate of plastic endothelial cells [112,113]. Macrophage-derived inflammatory signals can also act on VSMCs and other components of the calcifying lesion [12,115,116]. These observations provide a link between EndMT and the broader role of inflammation in amplifying vascular calcification.

### 3.4. Level IV. Amplification and Memory

#### 3.4.1. Inflammation

Inflammation and vascular calcification are closely coupled, but their relationship is bidirectional. Cytokine signaling can act upstream of mineral deposition by making vascular cells more permissive to osteogenic reprogramming and mineral growth. Conversely, once calcium-phosphate mineral forms, mineral particles can activate inflammatory pathways and further amplify the calcifying response [12,61,63,115,116]. This reciprocity is especially evident in atherosclerosis, where macrophages and mineral coexist within the same lesion, but it also emerges in CKD after systemic mineral stress has initiated medial calcification.

TNF-α provided some of the earliest evidence that inflammation can actively alter the mineralizing phenotype. It increases alkaline phosphatase and calcification in susceptible vascular cells, while monocytes and macrophages enhance vascular-cell mineralization through both direct contact and soluble mediators [12,115,116]. In diabetic LDLR-deficient mice, TNF-α neutralization reduces BMP2–MSX2–WNT signaling and aortic calcium despite persistent metabolic disease, directly linking inflammation to the developmental pathways described above [101]. Importantly, vascular calcification has also been demonstrated in other diabetic models, including Ins2^Akita/+^ mice, in which endothelial plasticity contributes to the calcifying phenotype [19,108]. Additional diabetic models implicate altered autophagic flux and vesicle-mediated inflammatory signaling in calcification [117,118], indicating that the procalcific vascular response in diabetes is not restricted to the LDLR-deficient model.

Macrophages can promote calcification without forcing VSMCs into a complete osteoblast-like state. Their secreted factors can increase BMP2 and reduce MGP, while RANKL further enhances macrophage production of TNF-α and IL-6, shifting the local environment toward mineralization [119,120].

Individual cytokines engage distinct downstream mechanisms. IL-6 trans-signaling recruits STAT3 and the histone demethylase JMJD2B to the *RUNX2* promoter, removes repressive H3K9me3 and stabilizes osteogenic transcription [121]. Macrophage-derived IL-1β also has a causal role: altering Rac signaling in macrophages changes IL-1β production and plaque calcification, while interruption of IL-1 signaling attenuates the phenotype [122]. IL-1β can additionally drive ROS-dependent p53–p21 senescence, followed by BMP2 induction and VSMC osteogenic conversion [123]. Inflammation can therefore influence calcification through transcriptional, epigenetic and senescence-associated routes.

The NLRP3 inflammasome adds a different dimension. NLRP3 activation accompanies experimental VSMC calcification, and its inhibition can suppress mineral deposition [124]. Yet NLRP3 should not be reduced to an IL-1β pathway. Under high-phosphate conditions, inhibition of NLRP3, caspase-1 or gasdermin D reduces calcification, whereas IL-1 receptor blockade does not [125]. In this setting, pyroptotic injury itself appears important. Membrane disruption and cellular debris may provide new mineral-binding surfaces independently of cytokine signaling.

The causal direction also runs from mineral to inflammation. Basic calcium-phosphate crystals are phagocytosed by macrophages and induce TNF-α, IL-1β and IL-8 through PKC and MAP-kinase pathways [126]. Smaller hydroxyapatite particles are particularly inflammatory, emphasizing that mineral surface area and architecture matter as much as total calcium burden [127]. Calcium-phosphate nanocrystals are also internalized by VSMCs, dissolve within lysosomes and produce a large intracellular calcium load followed by cell death [128].

Calciprotein particles provide a physiologically relevant version of this interaction. Secondary CPPs induce VSMC oxidative stress, TNF-α production and calcification, while TNF/TNFR1 inhibition attenuates the response [63]. In macrophages, CPP uptake produces both NLRP3-dependent IL-1β and partly NLRP3-independent IL-1α release through lysosomal and ionic disturbances [65]. Thus, disturbed mineral metabolism can be translated directly into sterile innate immune activation.

Inflammation is consequently best viewed as an amplifier whose position changes during disease. Before mineralization, cytokines alter BMP–WNT signaling, RUNX2, senescence and extracellular mineral defenses; after mineral appears, crystals and CPPs become persistent inflammatory stimuli that injure VSMCs and activate macrophages. ROS provide an important link between these directions, because inflammatory cytokines increase oxidative stress while mineral particles generate ROS during uptake and intracellular processing.

#### 3.4.2. Oxidative Stress and Mitochondrial Dysfunction

Oxidative stress is also bidirectionally related to vascular calcification. ROS can act upstream by promoting procalcific signaling and osteogenic reprogramming, but they can also arise downstream from inflammation, mineral-particle uptake and mitochondrial dysfunction [17,63,129,130]. In this setting, oxidative stress can further amplify calcification rather than simply reflect tissue injury. Mitochondria add another dimension because they regulate not only redox balance, but also ATP production, calcium homeostasis and organelle quality control.

A direct link between redox signaling and osteogenic reprogramming was demonstrated when H_2_O_2_ induced RUNX2, alkaline phosphatase and VSMC mineralization through AKT; RUNX2 depletion prevented the response [17]. Mitochondrial superoxide can similarly activate NF-κB, and reducing mitochondrial ROS suppresses calcification both in cultured VSMCs and in experimental CKD [129]. Oxidative stress can also appear before marked hyperphosphatemia: in CKD models, NOX1-dependent ROS and ERK activation accompany early RUNX2 induction and are attenuated by antioxidant intervention [130].

Yet mineral can lie upstream of ROS. Secondary calciprotein particles provoke oxidative stress and TNF-α production in VSMCs [63]. This provides a direct route by which established mineral can reinforce redox-dependent procalcific signaling. This distinction is important because much of the experimental literature uses phosphate-rich media in which cellular signaling and extracellular mineral generation occur simultaneously.

Mitochondrial quality control also contributes to the calcifying phenotype. Calcifying VSMCs show altered mitochondrial membrane potential, increased DRP1-associated fission and apoptotic signaling [131]. Interventions that preserve mitochondrial integrity reduce mineralization [131]. Lactate provides a more mechanistic example: it suppresses BNIP3-dependent mitophagy while promoting mitochondrial dysfunction, ROS production and calcification, and later work linked this defect to NR4A1–DNA-PKcs–p53-dependent DRP1 activation [131,132]. Conversely, restoring the PGC-1α–SIRT3–SOD2 axis preserves mitochondrial antioxidant capacity and suppresses vascular calcification [133].

The most informative connection between mitochondria and mineralization, however, is not necessarily ROS. In the *Lmna*^G609G/+^ model of Hutchinson–Gilford progeria, mitochondrial dysfunction reduces ATP production while TNAP activity increases [21]. Extracellular ATP is the substrate from which ENPP1 generates pyrophosphate; reduced ATP therefore limits pyrophosphate production precisely as increased TNAP accelerates its degradation. Severe arterial calcification develops despite unchanged circulating calcium and phosphate, and pyrophosphate replacement strongly suppresses it [21]. Here, mitochondrial failure alters extracellular mineral chemistry directly rather than acting exclusively through an osteogenic transcriptional pathway.

Subsequent studies reinforced this metabolic connection. ATP supplementation, particularly when combined with inhibition of extracellular ATP and pyrophosphate degradation, reduced calcification and prolonged survival in progeroid mice [134]. Magnesium improved mitochondrial respiration, NADPH-dependent antioxidant capacity and ATP availability while reducing vascular mineralization and extending lifespan [135]. These experiments link mitochondrial bioenergetics, redox defense and extracellular mineral inhibition within the same disease model.

Mitochondrial dysfunction should therefore be viewed as a loss of vascular resilience. A failing mitochondrion generates excess ROS, provides less ATP for pyrophosphate synthesis, handles calcium poorly and becomes increasingly dependent on effective mitophagy. Mineral particles, inflammatory cytokines and metabolic stress can further damage the same organelle. Once this reciprocal injury is established, mitochondrial dysfunction no longer needs to be the initiating event to accelerate disease.

#### 3.4.3. Cellular Senescence

Cellular senescence provides a mechanism by which transient vascular injury can become persistent. Senescent VSMCs remain metabolically active despite durable proliferative arrest and acquire altered redox, matrix and secretory functions. The relevance of senescence to calcification therefore lies less in a complete osteoblast-like conversion than in the persistence of a calcification-prone phenotype involving altered redox, matrix, secretory and mineral-regulatory functions [18,136]. Replicatively senescent human VSMCs are markedly more susceptible to calcification than younger cells and show increased RUNX2, alkaline phosphatase and collagen I together with reduced MGP [18]. The phenotype is incomplete: classical osteoblast markers do not change uniformly, indicating that senescence creates a calcification-prone state rather than a faithful osteoblast conversion. Transcriptomic studies similarly reveal simultaneous changes in inflammatory, extracellular-matrix and mineral-regulatory genes [136].

Persistent DNA damage provides one of the strongest mechanistic links. During vascular aging, oxidative stress reduces ZMPSTE24-dependent lamin A processing, causing accumulation of prelamin A, nuclear abnormalities and premature senescence [137]. Forced prelamin A expression accelerates VSMC calcification through chronic ATM/ATR-associated DNA-damage signaling and generates a senescence-associated secretory phenotype (SASP) containing IL-6, BMP2 and osteoprotegerin. Conditioned signaling from these cells can induce osteogenic activity in otherwise uninjured neighboring progenitors [138]. Senescence can therefore propagate calcification susceptibility without requiring every cell to experience the original insult.

The DNA-damage response itself is context-dependent. Chronic unresolved ATM signaling can sustain the SASP, whereas successful ATM-dependent repair is protective. VSMC-specific loss of SIRT6 impairs DNA repair and aggravates vascular calcification, while SIRT6 overexpression is protective [139]. GATA6 reinforces the opposite state by interfering with SIRT6/ATM-mediated repair; its knockdown reduces both VSMC senescence and CKD-associated arterial calcification [140]. These studies indicate that defective genome maintenance can contribute causally to a durable procalcific phenotype; DNA damage in this setting is therefore not merely a passive marker of vascular aging.

Age-regulated microRNAs provide another layer of persistence. *miR-34a* deletion reduces VSMC senescence and medial calcification while preserving SIRT1 and Axl, and miR-34a rises before overt mineralization in experimental disease [141]. Senescent VSMCs subsequently transmit this state through an IL-6-rich SASP [142]. Uremic toxins and inflammatory cytokines can reach a similar endpoint: indoxyl sulfate drives oxidative stress, prelamin A accumulation and p53–p21 senescence [143], whereas IL-1β couples ROS-dependent senescence to BMP2 and calcification [123]. Endothelial cells can also relay senescence-associated mineral stress to VSMCs through altered extracellular vesicles [144,145].

Importantly, senescence does not require a strong conventional osteogenic program to increase calcification competence. SIRT7 loss, for example, enhances ROS, senescence and vascular calcification with comparatively modest effects on classical osteogenic differentiation [146]. The consequences of senescence can instead emerge through impaired mitochondrial function, loss of mineralization inhibitors, altered vesicle release, matrix remodeling and persistent inflammatory signaling.

The presence of p16, p21 or SA-β-gal alone should not be taken as proof of senescence either. These markers are individually nonspecific, and many acute calcification models do not establish irreversible growth arrest or a functional SASP. Direct evidence that selective elimination of senescent vascular cells reverses established calcification also remains comparatively limited. The strongest causal evidence still comes from manipulating pathways that generate or maintain the senescent state—prelamin A, miR-34a, SIRT6 and GATA6—rather than from senolysis itself.

Recent work further suggests that the molecular memory of mineral stress may begin before classical senescence is fully established. Prelamin A and CKD-associated mineral dysregulation cause early loss of the repressive heterochromatin marks H3K9me3 and H3K27me3, accompanied by inflammatory and osteogenic transcriptional changes. In experimental CKD and arteries from children receiving dialysis, heterochromatin loss was detectable before overt calcification, suggesting that chromatin reorganization may precede—and help establish—the persistent aged phenotype [147].

Senescence can therefore stabilize vascular calcification by allowing cells generated by mineral, inflammatory or oxidative stress to persist and continue altering neighboring cells, matrix and mineral defenses. This persistence provides a cellular basis for calcification memory.

#### 3.4.4. Epigenetic and Metabolic Reprogramming

A transient mineral, inflammatory or oxidative insult can subside while a calcification-prone phenotype persists. Epigenetic and post-transcriptional regulation provide plausible mechanisms for this cellular memory, but they are closely coupled to metabolism. DNA methyltransferases depend on S-adenosylmethionine (SAM) and are inhibited by S-adenosylhomocysteine (SAH), sirtuins require NAD^+^, histone acetylation draws on acetyl-CoA, and glycolytic lactate can modify chromatin. Metabolism therefore influences not only energy supply but also the regulatory state that VSMCs maintain.

CKD provides several examples. Indoxyl sulfate increases DNMT1/3A-dependent methylation of the *KLOTHO* promoter, reducing vascular Klotho expression and increasing calcification; demethylation partially reverses this phenotype [148]. A more direct metabolic–epigenetic link involves S-adenosylhomocysteine hydrolase (SAHH). Reduced SAHH activity raises SAH, alters DNMT3B-dependent methylation of the *H19* promoter and activates an H19–RUNX2 program, while perturbation of adenosine–AMPK–SIRT1 signaling provides a parallel metabolic route [149]. Thus, disruption of a single metabolic pathway can simultaneously affect DNA methylation and cellular energy sensing.

Non-coding RNAs, particularly microRNAs, provide an additional post-transcriptional layer of regulation across the calcification pathways described above. miR-125b decreases during VSMC calcification, and its experimental inhibition enhances mineralization partly through derepression of SP7 [150]. miR-133a similarly restrains osteogenic differentiation by directly targeting the *RUNX2* 3′UTR [151]. In experimental CKD, high phosphorus alters a broader network involving miR-29b, miR-133b and miR-211; mimics and inhibitors modify the calcification phenotype, linking mineral stress to miRNA-dependent control [152]. These observations support miRNAs as modifiers of calcification susceptibility rather than independent initiators of mineral deposition.

Histone regulation is equally context-dependent. Broad HDAC inhibition can enhance phosphate-induced alkaline phosphatase activity and calcification [153]. HDAC1 appears protective: its MDM2-dependent degradation accompanies mineralization, whereas preservation of HDAC1 suppresses it [154]. HDAC9 has the opposite effect. Human genetic association implicated the *HDAC9* locus in abdominal aortic calcification, and *Hdac9* deficiency reduced calcification in *Mgp*-deficient mice [155]. The biological effect of histone acetylation therefore depends on the enzyme and genomic context rather than on acetylation itself.

Inflammatory signaling can also reshape chromatin directly. IL-6/STAT3 recruits JMJD2B/KDM4B to the *RUNX2* promoter, removes repressive H3K9me3 and facilitates RUNX2 transcription; disrupting either STAT3 or JMJD2B attenuates the osteogenic response [121]. This provides a mechanistic route by which inflammatory exposure can outlast the initiating signal.

Metabolic reprogramming intersects with these processes at several levels. The emphasis here is on metabolic pathways with direct experimental links to vascular calcification rather than on descriptive metabolomic signatures. PDK4 promotes calcification while impairing mitochondrial respiration, mitochondria–ER contacts, lysosomal acidification and autophagic flux, whereas PDK4 depletion restores organelle function and reduces mineralization [156]. Yet indiscriminate glycolytic inhibition can worsen oxidative injury and apoptosis, indicating that increased glycolytic dependence may also represent an adaptation required for survival. More selective manipulation identifies PFKFB3 as a procalcific node: its inhibition reduces glycolytic flux, lactate production and experimental calcification, whereas pyruvate or lactate partially restores the phenotype [157].

Lactate is especially relevant because it connects metabolic flux directly to chromatin. *Nr4a3* deficiency reduces calcification in two mouse models and decreases ALDOA/PFKL expression, lactate production and histone lactylation. Integrated chromatin and transcriptional analyses identified *PHOSPHO1* as a downstream effector, and manipulation of PHOSPHO1 altered the NR4A3-dependent calcification phenotype [158]. This provides unusually direct evidence linking glycolytic regulation, histone modification and mineralization.

A more recent study extends this connection to RNA regulation. H3K18 lactylation was reported to enhance *ALKBH5* transcription; ALKBH5 then stabilized *RUNX2* mRNA through m^6^A demethylation, while RUNX2 increased LDHA expression and lactate production, generating an H3K18la–ALKBH5–RUNX2–LDHA feedback loop [159]. The mechanism integrates glycolysis, histone lactylation and RNA stability in a single circuit, but its recent origin warrants independent confirmation.

These studies suggest that epigenetic, post-transcriptional and metabolic regulation primarily determine the persistence and responsiveness of the calcification-prone cell state. They provide a molecular basis for hysteresis: previous mineral or inflammatory exposure can alter how VSMCs respond to subsequent insults even after the original stimulus has diminished. As metabolic compensation becomes insufficient, however, maintenance of this state increasingly depends on organelle quality control and protein homeostasis, linking metabolic–epigenetic reprogramming to autophagy, lysosomal function and ER stress.

### 3.5. Level V. Stress Containment and Homeostatic Failure

#### 3.5.1. Autophagy and ER Stress

Autophagy and the unfolded protein response are fundamentally homeostatic systems. Their presence in calcifying arteries therefore does not imply that they initiate disease. Both are recruited when VSMCs encounter mineral, oxidative, inflammatory or metabolic stress; pathology emerges when these responses can no longer restore intracellular homeostasis.

Autophagy illustrates this distinction particularly well. High phosphate activates autophagy in VSMCs, yet inhibition of *ATG5* or pharmacological blockade of autophagy increases rather than decreases calcification. Conversely, stimulation of autophagy is protective [20]. Importantly, autophagy inhibition increases the release and alkaline phosphatase activity of matrix vesicles even when apoptosis is suppressed. Autophagy therefore appears to contain calcification partly by preventing stressed intracellular material from being redirected into extracellular nucleation-competent vesicles.

The relevant variable is autophagic flux, not simply autophagosome abundance. Increased LC3-II can reflect either efficient autophagy or failure of lysosomal degradation. This distinction becomes evident in diabetic VSMCs, where periostin increases LC3-II and Beclin-1 but also causes p62 accumulation and defective autophagosome–lysosome fusion; restoring flux reduces calcification [117]. PDK4 similarly disrupts mitochondria–ER contacts, mitochondrial respiration, lysosomal acidification and TFEB-dependent lysosomal regulation, producing a genuine block in autophagic degradation [156]. Notably, global glycolytic inhibition worsens oxidative injury and calcification in this model, reinforcing the broader principle that cellular adaptations induced during calcification are not necessarily themselves pathogenic.

Mineral can also create the autophagic defect rather than merely result from it. Nano-hydroxyapatite resembling mineral recovered from human calcified arteries is internalized by VSMCs and delivered to lysosomes, where it impairs V-ATPase-dependent acidification and blocks autophagic flux [160]. Re-acidifying lysosomes restores degradation and reduces mineralization. When lysosomal processing fails, accumulated autophagic compartments are redirected into calcium-, phosphate- and alkaline-phosphatase-containing exosomes that promote further extracellular mineral deposition. Thus, existing mineral can disable the pathway that would otherwise help contain it, converting impaired intracellular clearance into extracellular propagation.

Autophagy can similarly restrain inflammatory cargo. In diabetic calcification, vesicular structures released by VSMCs carry IL-1β and promote calcification in recipient cells; rapamycin reduces this vesicular IL-1β release, whereas chloroquine enhances it [118]. Autophagic competence therefore influences not only intracellular quality control but also what a stressed VSMC exports into its surroundings.

Endoplasmic reticulum stress has been implicated in vascular calcification across several experimental settings, including lipid stress, inflammatory signaling and CKD [161,162,163,164,165]. The unfolded protein response initially reduces protein-folding burden and attempts to restore ER homeostasis. With persistent stress, however, PERK–eIF2α signaling sustains ATF4 and eventually CHOP, shifting the response toward osteogenic reprogramming and cell injury. Saturated fatty acids provide strong causal evidence: stearate activates PERK–eIF2α, ATF4 and CHOP, whereas ATF4 depletion markedly suppresses osteogenic differentiation and mineralization [166]. Smooth-muscle-specific loss of SCD1/2 later identified fully saturated phosphatidic acids as important upstream lipid species, linking membrane composition itself to ER stress and vascular calcification [167].

Inflammation converges on the same pathway. TNF-α activates PERK–eIF2α–ATF4–CHOP in VSMCs, and depletion of PERK, ATF4 or CHOP suppresses the resulting osteogenic phenotype. Neutralization of TNF-α or chemical relief of ER stress also reduces calcification in CKD mice [161]. CHOP is particularly important at the transition from adaptation to failure: genetic *Chop* deficiency protects against CKD-associated vascular calcification and cell death [162], whereas smooth-muscle-specific CHOP expression produces severe apoptosis and medial calcification when combined with CKD [163]. These reciprocal manipulations make the ATF4–CHOP axis considerably more than a marker of stressed calcifying cells.

Autophagy and ER stress therefore converge on a question of stress containment. A VSMC with functional lysosomes can remove damaged proteins, organelles and mineral-containing compartments; an adaptive unfolded protein response (UPR) can temporarily reduce proteostatic burden. Persistent mineral, lipid or inflammatory stress eventually overwhelms these systems. Lysosomal flux fails, vesicular export increases and prolonged ATF4–CHOP signaling shifts from adaptation toward cellular injury.

This transition also clarifies their position within the broader calcification process. Neither autophagic dysfunction nor ER stress is required for calcium phosphate to form in a sufficiently permissive extracellular environment. Their importance lies in determining whether a living vessel can contain that mineral pressure. Once proteostasis and degradative capacity collapse, however, the alternatives narrow: the stressed VSMC increasingly progresses toward irreversible membrane and mitochondrial injury and, ultimately, death. The next step is therefore to examine regulated cell death—not as a single pathway, but as several distinct mechanisms by which cellular failure creates new substrates for vascular mineralization.

#### 3.5.2. Regulated Cell Death

Regulated cell death represents the point at which failure of cellular homeostasis is converted most directly into extracellular mineralization. A dying VSMC loses the ability to maintain pyrophosphate, matrix inhibitors and membrane homeostasis while simultaneously leaving behind phospholipid-rich structures capable of concentrating calcium. Cell death therefore has a dual effect: it removes a mineral-resistant cell and creates a potential mineralization nidus.

This relationship is best established for apoptosis. In human VSMCs, apoptosis precedes spontaneous calcification; caspase inhibition reduces mineral deposition, whereas Fas-induced apoptosis increases it almost tenfold. Isolated apoptotic bodies concentrate calcium and contain crystalline mineral, providing direct evidence that products of cell death can nucleate calcification [13]. Their exposed phosphatidylserine offers a negatively charged membrane surface with high affinity for Ca^2+^, closely resembling the nucleation chemistry of calcifying matrix vesicles.

Persistence of these structures is as important as their generation. Acetylated LDL increases VSMC calcification without increasing apoptosis itself, instead impairing phagocytic removal and causing apoptotic bodies to accumulate [164]. Conversely, fetuin-A reduces apoptosis, decreases the mineralizing capacity of released vesicles and promotes their clearance [56]. Thus, defective efferocytosis can convert an otherwise transient episode of cell death into persistent extracellular nucleation.

Apoptosis is nevertheless only one source of membrane-bound mineral nuclei. Living VSMCs also release calcifying matrix vesicles, particularly under elevated calcium and phosphate conditions [27]. Thus, inhibition of apoptosis can reduce mineralization [13], but does not eliminate the independent contribution of matrix vesicles released by viable VSMCs [27]. These findings indicate that apoptotic bodies and matrix vesicles represent distinct cellular routes to extracellular mineral nucleation.

The relationship also runs in reverse. Small calcium-phosphate crystals are internalized by human VSMCs and dissolve within acidic intracellular compartments, producing a rapid rise in cytosolic calcium followed by cell death [128]. Mineral can therefore generate the death that supplies further nucleation surfaces. ER stress strengthens this transition: ATF4–CHOP signaling couples prolonged proteostatic failure to apoptosis and calcification, and genetic manipulation of CHOP changes vascular mineralization in CKD models [162,163,165]. Likewise, high phosphate can suppress the Gas6–Axl survival pathway, increasing apoptosis and calcification [168], although—as discussed earlier—such experiments cannot fully separate soluble phosphate signaling from the effects of mineral generated in phosphate-rich medium.

Pyroptosis introduces a more inflammatory form of membrane failure. Calcifying VSMCs can activate NLRP3, caspase-1 and gasdermin D, and pharmacological inhibition of this pathway reduces experimental mineral deposition [169]. More direct dissection showed that NLRP3 or caspase-1 inhibition suppresses high-phosphate calcification whereas IL-1 receptor blockade does not [125]. The implication is that gasdermin-mediated cell disruption itself may contribute to mineralization independently of canonical IL-1β signaling. A pyroptotic cell releases membrane fragments and intracellular material directly into an extracellular environment already rich in calcium and phosphate.

Ferroptosis provides a chemically distinct route. High calcium and phosphate suppress the SLC7A11–glutathione–GPX4 antioxidant system, while ferrostatin-1 reduces calcification in cultured VSMCs, human and rat arterial rings, vitamin D-loaded mice and CKD rats. Conversely, depletion of SLC7A11 or inhibition of GPX4 increases mineralization [170]. These complementary interventions provide stronger mechanistic evidence than changes in lipid ROS or GPX4 expression alone that ferroptotic susceptibility contributes to vascular calcification. Vitamin D loading therefore also provides an in vivo experimental context in which vascular calcification can be studied, although these data do not establish VDR signaling itself as an independent calcification mechanism [170].

Atherosclerotic lipid stress can engage the same pathway. The oxidized phospholipid POVPC increases lipid peroxidation, mitochondrial dysfunction and VSMC calcification, all attenuated by ferrostatin-1 [171]. Increased SLC39A14/SLC39A8-dependent iron accumulation similarly promotes ferroptotic features and calcification, whereas transporter inhibition or iron chelation is protective [172]. The precise way in which ferroptotic death accelerates mineral growth remains less firmly established than for apoptotic bodies, however. Ferroptosis clearly changes calcification susceptibility; whether ferroptotic membrane remnants themselves constitute major nucleation sites in vivo still requires direct demonstration.

Recent evidence suggests that death programs may also overlap. A 2026 study reported simultaneous activation of apoptotic, pyroptotic and necroptotic machinery in CKD-associated vascular calcification and proposed a PANoptotic state regulated by cartilage oligomeric matrix protein and AT1R–β-arrestin-2 signaling [173]. The finding is intriguing, particularly because inhibition of β-arrestin-2 attenuated both cell-death signaling and calcification, but PANoptosis in vascular mineralization remains an emerging concept rather than an established mechanism.

The mechanistic importance of cell death ultimately extends beyond the identity of the execution pathway. Apoptosis packages membranes into bodies; pyroptosis and necroptosis rupture the cell; ferroptosis destroys membranes through lipid peroxidation. Each process changes the abundance and composition of extracellular material exposed to a supersaturated calcium-phosphate environment. At the same time, every lost VSMC represents loss of ATP production, pyrophosphate generation, mineralization inhibitors, matrix maintenance and clearance capacity.

Regulated cell death therefore closes the mechanistic circuit of vascular calcification. Calcium and phosphate create mineral pressure; failure of inhibitors permits nucleation; particles and vesicles propagate mineral; matrix remodeling retains it; cellular reprogramming, inflammation and metabolic stress amplify the response; and failure of proteostasis and organelle quality control ultimately kills the cells that had been restraining mineralization. Their remnants then provide new surfaces for crystal growth, while the mineral itself injures the surviving cells. What begins as disturbed extracellular chemistry can thus become a self-sustaining tissue process in which mineral formation and cellular failure continually regenerate one another.

## 4. Functional Coupling Across the Five Levels

The five functional levels are not independent compartments but parts of a coupled system in which extracellular mineral chemistry and vascular cell biology progressively reinforce one another (Figure 4). At the interface between Levels I and II, the balance between mineralizing pressure and inhibitory capacity determines whether calcium phosphate emerges, while fetuin-A, extracellular vesicles and extracellular matrix architecture influence how nascent mineral is packaged, where it nucleates and whether it is retained [9,14,15,25,73,78].

Mineral organization then changes the environment experienced by vascular cells. Calcium-phosphate particles, mineralizing vesicles and altered matrix are not inert consequences of calcification; they provide biochemical and mechanical signals capable of modifying vascular cell phenotype. VSMCs can lose components of their contractile identity and acquire RUNX2-dependent osteochondrogenic programs, while BMP–WNT signaling stabilizes and propagates these responses through the vessel wall [22,100]. These cellular changes can in turn increase alkaline phosphatase activity and alter the composition and mineralizing potential of extracellular vesicles [71,74,100]. Taken together, these observations support a reciprocal relationship between mineral formation and cellular reprogramming: extracellular mineral can promote phenotypic change in vascular cells [26,63,76,86], while reprogrammed cells can subsequently generate a more mineralization-prone extracellular environment [71,74,100]. The mechanisms grouped in Level IV provide a means by which this response becomes persistent. Mineral-containing particles can induce inflammatory and oxidative responses, while inflammatory cytokines promote osteogenic signaling and alter extracellular mineral defenses [63]. Oxidative stress and mitochondrial dysfunction also connect cellular injury directly back to mineral regulation. Reduced mitochondrial ATP production limits the substrate available for extracellular pyrophosphate synthesis, thereby providing a direct route by which cellular metabolic dysfunction can weaken mineral inhibition [21]. Thus, dysfunction arising downstream of mineral formation can feed back to Level I and increase mineral permissiveness. Senescence and epigenetic–metabolic reprogramming provide additional persistence by stabilizing altered cellular states after the initiating mineral, inflammatory or metabolic disturbance has diminished. Level IV therefore introduces biological memory into the system: previous injury alters how the vessel responds to subsequent mineral stress even after the initiating stimulus has diminished.

Level V determines whether these stresses can still be contained. Autophagy and the unfolded protein response initially protect the cell by maintaining organelle and protein homeostasis, and effective autophagic flux limits procalcific vesicular release [20]. Persistent mineral, oxidative, metabolic or proteostatic stress can nevertheless exceed this adaptive capacity. Regulated cell death then has consequences that extend beyond the loss of the individual VSMC. Apoptotic bodies provide phospholipid-rich calcium-binding surfaces capable of nucleating mineral directly [13]. Loss of viable VSMCs can also weaken local mineral protection, since extracellular ATP provides the substrate for ENPP1-dependent pyrophosphate generation [24,45]. In parallel, impaired clearance allows apoptotic bodies to persist and provides additional extracellular nucleation sites [56,168]. Cellular failure can therefore both generate new mineralization substrates and weaken the mechanisms that normally limit their persistence. This last step closes the functional circuit summarized in Figure 4. Cellular stress and death generate vesicular debris and new nucleation surfaces that return the process to Level II, while loss of metabolic and inhibitory capacity feeds back toward Level I. Vascular calcification can consequently become self-sustaining once mineral formation begins to damage the systems responsible for containing it.

The dominant entry point into this circuit is likely to differ between disease settings: CKD-associated medial calcification may enter predominantly through mineral dysregulation and failure of extracellular inhibition, whereas atherosclerotic lesions may engage inflammatory, matrix and cellular pathways before extensive mineral is detectable. Monogenic vascular calcification disorders provide a complementary example: ENPP1 deficiency illustrates how severe loss of extracellular pyrophosphate protection can drive arterial mineralization through Level I [15]. The framework does not imply that the same initiating mechanism operates in every individual or disease context; rather, different combinations of systemic and local conditions may engage the five functional levels to different degrees. It is therefore intended to organize shared mechanisms of vascular calcification rather than to predict individual disease susceptibility.

## 5. Conclusions and Perspectives

Vascular calcification is increasingly difficult to accommodate within either of its traditional extremes. It is neither the passive precipitation of calcium phosphate onto an inert arterial wall nor the simple conversion of vascular cells into osteoblasts. Calcium and phosphate can generate mineral independently of transcription, whereas living vascular cells profoundly determine whether that mineral is inhibited, packaged, retained, amplified or cleared [25,174]. Conversely, osteogenic reprogramming is functionally important—smooth-muscle-specific loss of RUNX2 reduces vascular calcification—but it acts within an extracellular environment whose mineral chemistry remains indispensable [22]. The distinction is more than semantic. It places failure of mineral containment, rather than any single signaling pathway, at the center of the disease.

Several questions now become more important than identifying additional procalcific pathways. Foremost is temporal causality in human disease. Much of the mechanistic literature relies on models in which phosphate, calcium, inflammation or oxidative stress are imposed at concentrations that simultaneously alter extracellular mineral chemistry and intracellular signaling. Distinguishing responses to soluble ions from responses to newly formed nanoparticles remains essential. Equally important is determining how the relative contribution and causal order of these mechanisms differ between intimal atherosclerotic calcification, CKD-associated medial calcification, diabetes and genetic disorders of mineral inhibition. The potential therapeutic targets, translational readouts and principal limitations associated with each functional level are summarized in Table S4.

The framework also suggests that therapeutic efficacy may depend on when the system is targeted. Interventions that reduce mineral pressure or restore extracellular inhibition may be most effective before extensive nucleation and tissue remodeling have occurred. Experimental restoration of pyrophosphate strongly suppresses vascular mineralization, including in settings of profound genetic or metabolic susceptibility [21,42,43,44]. Once mineralized matrix, senescent cells, inflammatory signaling and altered metabolic states have accumulated, however, correcting a single downstream pathway may be insufficient. At that stage, successful treatment may require simultaneous restoration of mineral inhibition and interruption of the cellular feedbacks that maintain the calcifying niche.

The framework may also help distinguish mineral burden from mineral architecture. In atherosclerotic lesions, extracellular-vesicle-derived mineral can initially appear as dispersed microcalcifications that subsequently aggregate and become incorporated into larger deposits [78]. Mineral size is biologically relevant: smaller hydroxyapatite particles have a greater inflammatory effect than larger particles, and calcium-phosphate nanocrystals can be internalized by VSMCs and induce lysosomal calcium loading and cell death [127,128]. Microcalcification and macrocalcification should therefore not be regarded simply as different amounts of the same material. Levels I and II influence where mineral nucleates, its packaging and growth, whereas Levels III–V determine how vascular cells respond to the resulting mineral architecture. However, the present framework does not imply that microcalcification is invariably destabilizing or that macrocalcification is invariably protective; the clinical consequences also depend on lesion location, composition and disease context.

An important therapeutic distinction is therefore between preventing new mineral deposition and removing established vascular mineral. Most interventions discussed in this review have been shown to prevent or slow calcification rather than to reverse mature deposits. Once mineralized matrix and self-reinforcing inflammatory, metabolic and stress-response pathways are established, correction of a single mechanism may be insufficient. Stage-specific or combined approaches that restore mineral inhibition while interrupting cellular amplification may therefore be required, although this remains to be demonstrated clinically.

A major priority is therefore to move from cataloging mechanisms toward establishing causal order, reversibility and therapeutic windows. Experimental systems should increasingly distinguish ion concentrations from particle formation and measure mineral composition alongside cell phenotype. They should also measure autophagic flux and establish the functional contribution of specific regulated cell-death pathways rather than relying on isolated markers, while distinguishing interventions that prevent new mineralization from those that remove established deposits. Human tissue studied across defined stages of disease will be particularly important for deciding which mechanisms identified in experimental models dominate in vivo.

The value of the five-level framework is therefore not to impose a fixed sequence, but to identify where causal control is exerted and where the process may remain therapeutically tractable. Vascular calcification becomes self-sustaining when mineral formation compromises the mechanisms that normally restrain it. Breaking this reciprocal coupling may ultimately be more important than targeting any individual component of the calcification program.

## Figures and Tables

**Figure 1:**
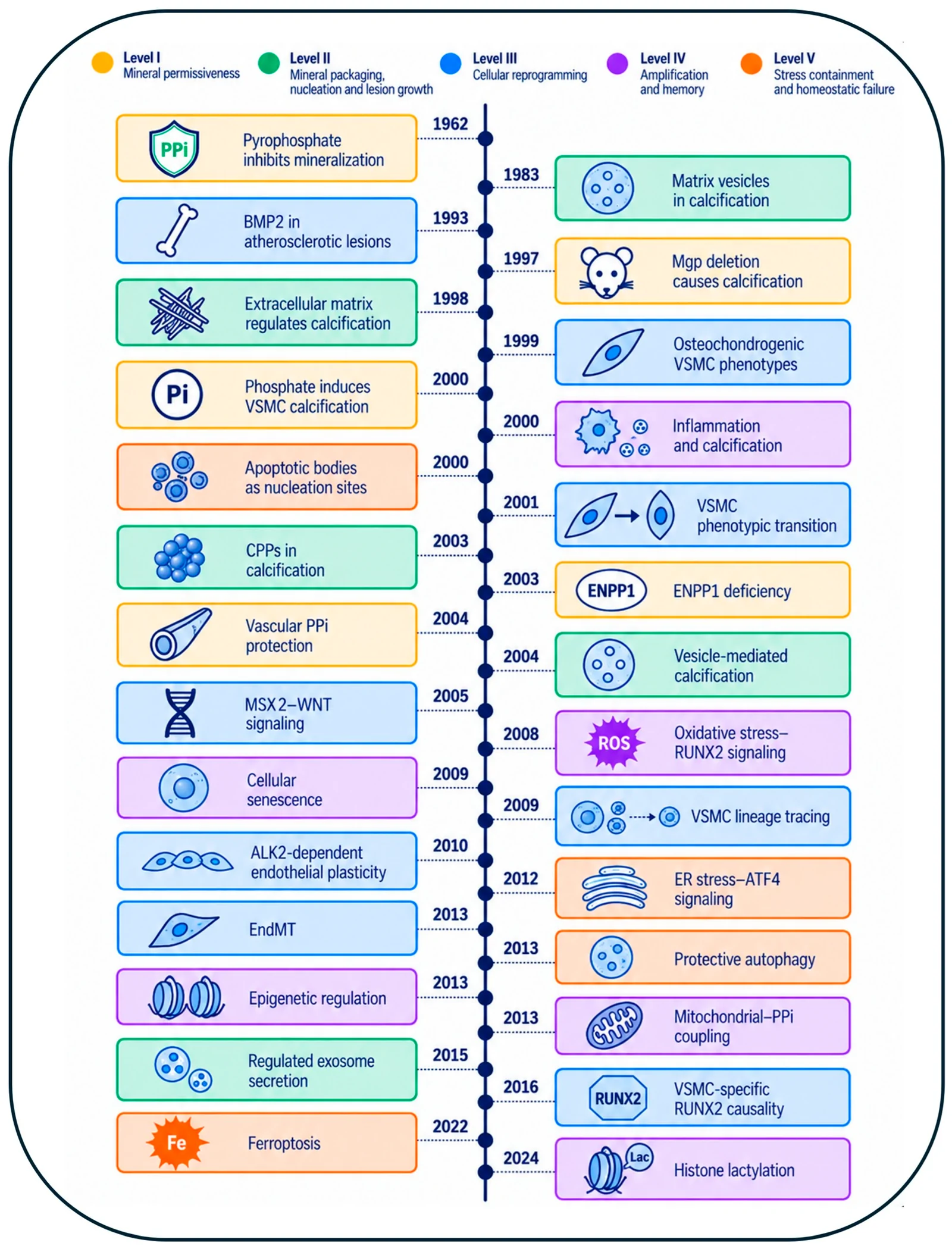
Historical perspective of vascular calcification. The timeline highlights key experimental and clinical observations that shaped the current view of vascular calcification. These milestones span the discovery of endogenous mineralization inhibitors, matrix vesicles and calciprotein particles, the emergence of osteogenic and inflammatory mechanisms, and later work on cellular stress, metabolic regulation and regulated cell death. Together, they illustrate how the field evolved from a largely physicochemical view of mineral deposition toward a broader model that integrates mineral handling, cellular reprogramming, amplification and failure of vascular homeostasis. Dates correspond to publication years of representative primary studies; further historical detail is provided in Table S1.

**Figure 2:**
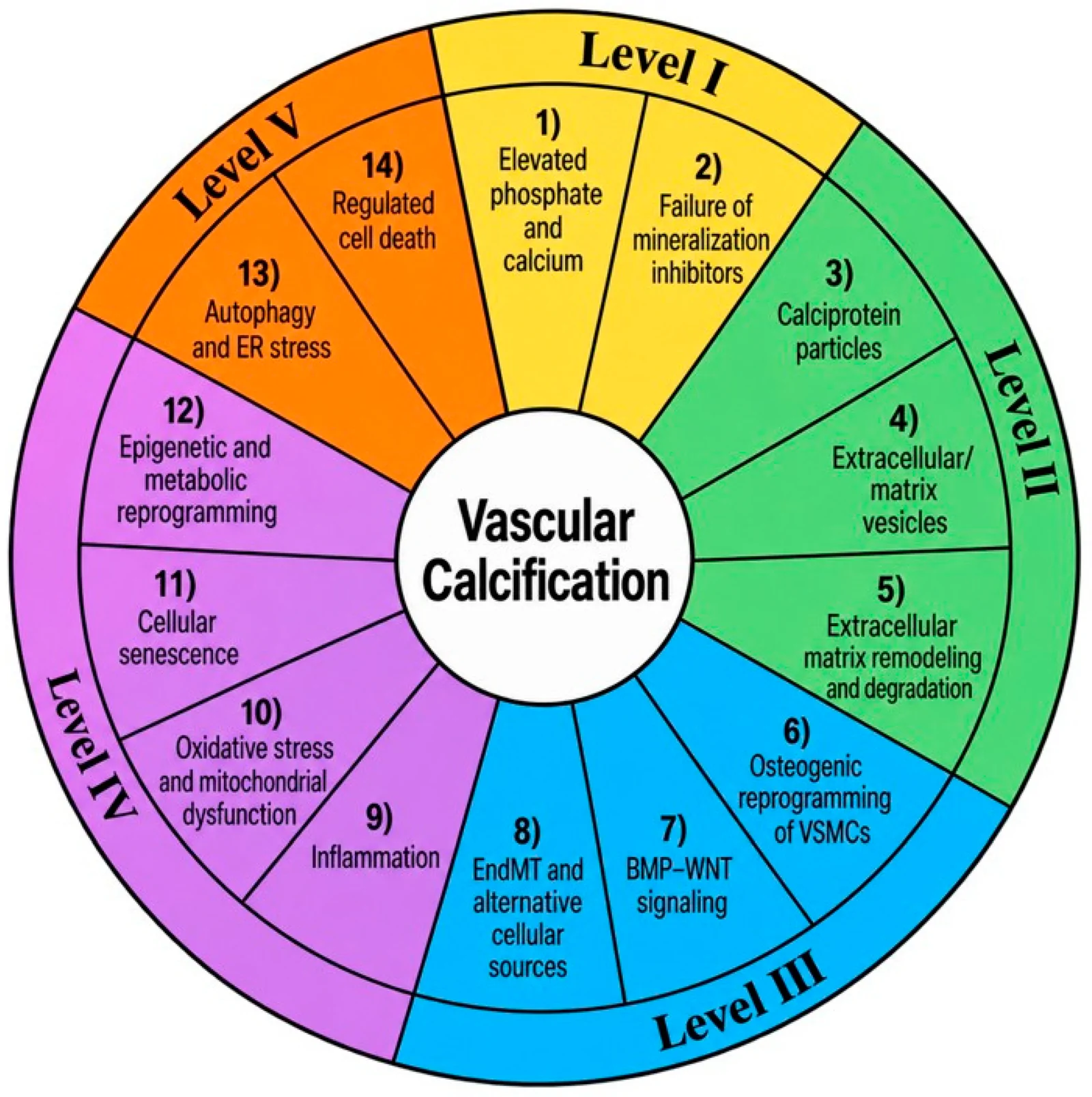
Hierarchical organization of vascular calcification mechanisms. Fourteen mechanisms are grouped into five functional levels according to their predominant causal role. Level I includes elevated phosphate and calcium and failure of mineralization inhibitors; Level II comprises calciprotein particles, extracellular/matrix vesicles, and extracellular matrix remodeling and degradation; Level III includes osteogenic reprogramming of VSMCs, BMP–WNT signaling, and EndMT and alternative cellular sources; Level IV comprises inflammation, oxidative stress and mitochondrial dysfunction, cellular senescence, and epigenetic and metabolic reprogramming; and Level V includes autophagy and ER stress and regulated cell death. The arrangement reflects functional organization rather than a fixed temporal sequence.

**Figure 3:**
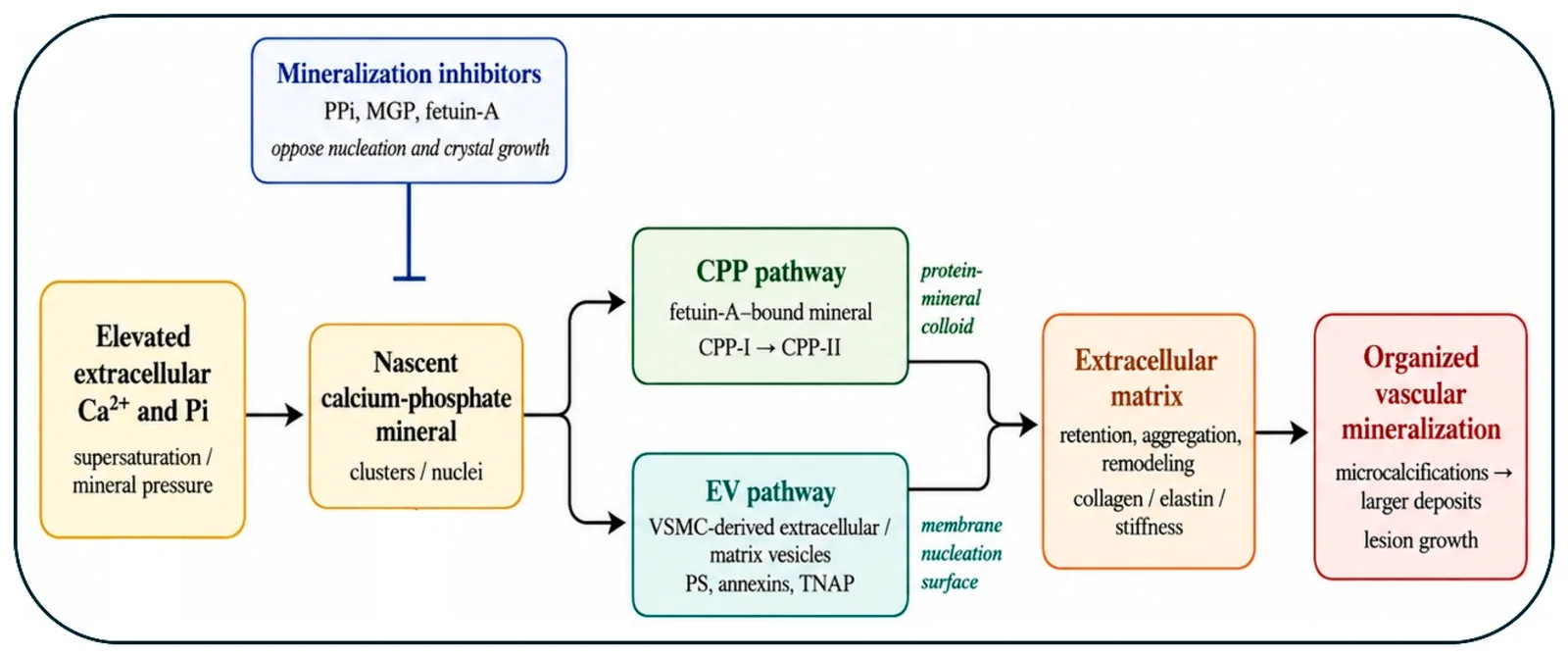
Extracellular determinants of vascular mineral nucleation and growth. Elevated extracellular Ca^2+^ and Pi increase mineral pressure and favor formation of nascent calcium-phosphate clusters. Mineralization inhibitors, including inorganic pyrophosphate (PPi), matrix Gla protein (MGP) and fetuin-A, oppose nucleation and crystal growth. Nascent mineral is subsequently handled through calciprotein particles (CPPs) and extracellular vesicle (EV) pathways, which differ in origin but both contribute to mineral organization and retention. Extracellular matrix composition and remodeling influence aggregation, persistence and growth of mineral deposits, leading from microcalcifications to larger vascular lesions. PS, phosphatidylserine; TNAP, tissue-nonspecific alkaline phosphatase.

**Figure 4:**
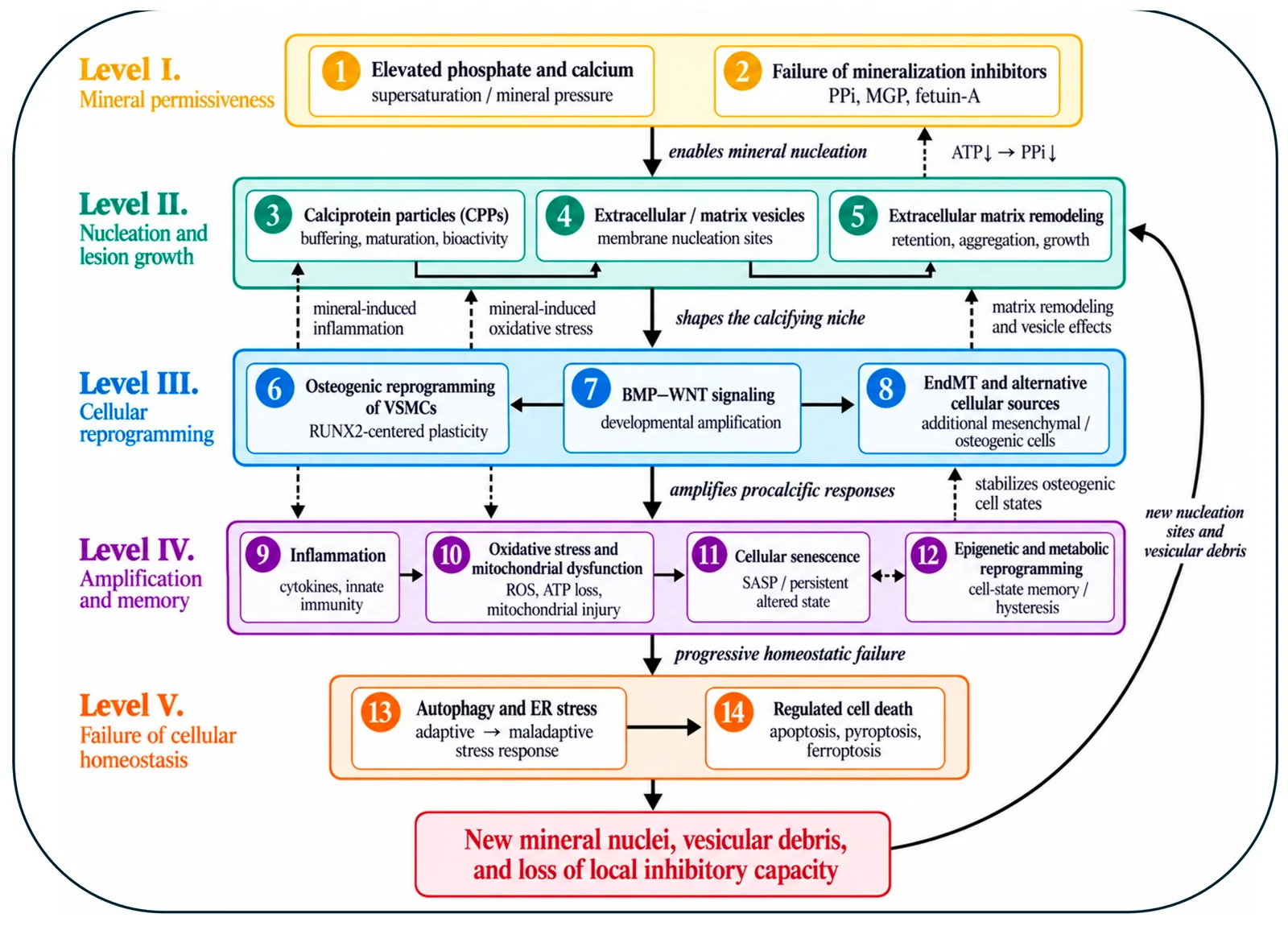
Functional coupling across the five levels of vascular calcification. The diagram summarizes the predominant causal relationships and reinforcing feedbacks linking mineral permissiveness, nucleation and lesion growth, cellular reprogramming, amplification and memory, and failure of cellular homeostasis. Solid arrows indicate predominant causal flow. Dashed arrows indicate specific cross-level feedbacks, including mineral/CPP-induced sterile inflammation and oxidative stress, mitochondrial ATP loss leading to reduced PPi-dependent mineral inhibition, and stabilization of osteogenic cell states by persistent epigenetic and metabolic reprogramming. Cellular failure generates vesicular debris and new nucleation surfaces that feed back to mineral nucleation and growth. The scheme represents functional coupling rather than an obligatory temporal sequence.

**Table 1:** Functional organization of the major mechanisms of vascular calcification.

Functional Level	Functional Question	Predominant Role
I	Can a mineral phase form?	Determines whether the extracellular environment can sustain calcium-phosphate mineralization.
II	Where and how does mineral nucleate, organize and persist?	Controls mineral packaging, nucleation surfaces, retention and lesion expansion.
III	How do vascular cells respond to and reshape the calcifying environment?	Alters cell identity and behavior, reinforcing tissue mineralization.
IV	How does the response become self-sustaining?	Creates positive feedback and persistent calcification-prone cellular states.
V	Can cellular damage be contained, or does homeostatic failure generate new calcifying substrates?	Determines recovery versus failure, vesicular release, cell death and renewed mineral nucleation.

## Data Availability

No new data were created or analyzed in this study. Data sharing is not applicable to this article.

## Supplementary Materials

The following supporting information can be downloaded at https://www.mdpi.com/article/10.3390/biology15191708/s1: Table S1: Historical Emergence and Mechanistic Consolidation of the Major Pathways of Vascular Calcification; Table S2: Disease context, vascular compartment and representative experimental models supporting the five-level framework; Table S3: Functional assignment, cross-level interactions, supporting evidence and principal limitations of the 14 mechanisms included in the five-level framework; Table S4: Potential therapeutic and translational implications of the five functional levels of vascular calcification.

## Abbreviations

ABCC6	ATP-binding cassette subfamily C member 6
AKT	protein kinase B
ALKBH5	AlkB homolog 5, RNA demethylase
ATF4	activating transcription factor 4
ATP	adenosine triphosphate
BMP	bone morphogenetic protein
BMP2	bone morphogenetic protein 2
BNIP3	BCL2 interacting protein 3
CHOP	C/EBP homologous protein
CKD	chronic kidney disease
CPP	calciprotein particle
CPP-I	primary calciprotein particle
CPP-II	secondary calciprotein particle
DNMT	DNA methyltransferase
DRP1	dynamin-related protein 1
EndMT	endothelial-to-mesenchymal transition
ENPP1	ectonucleotide pyrophosphatase/phosphodiesterase 1
ER	endoplasmic reticulum
ERK	extracellular signal-regulated kinase
EV	extracellular vesicle
FGF23	fibroblast growth factor 23
GPX4	glutathione peroxidase 4
GRP	Gla-rich protein
HDAC	histone deacetylase
IL	interleukin
IL-1β	interleukin-1 beta
IL-6	interleukin-6
KLF4	Krüppel-like factor 4
LDHA	lactate dehydrogenase A
MGP	matrix Gla protein
MMP	matrix metalloproteinase
NADPH	reduced nicotinamide adenine dinucleotide phosphate
NF-κB	nuclear factor kappa B
NLRP3	NLR family pyrin domain containing 3
NOX1	NADPH oxidase 1
NR4A1	nuclear receptor subfamily 4 group A member 1
NR4A3	nuclear receptor subfamily 4 group A member 3
PDK4	pyruvate dehydrogenase kinase 4
PERK	protein kinase R-like endoplasmic reticulum kinase
PFA	phosphonoformic acid
PFKFB3	6-phosphofructo-2-kinase/fructose-2,6-bisphosphatase 3
PGC-1α	peroxisome proliferator-activated receptor gamma coactivator 1-alpha
PHOSPHO1	phosphoethanolamine/phosphocholine phosphatase 1
Pi	inorganic phosphate
PPi	inorganic pyrophosphate
PiT-1	type III sodium-dependent phosphate transporter 1
PiT-2	type III sodium-dependent phosphate transporter 2
ROS	reactive oxygen species
RUNX2	runt-related transcription factor 2
SAH	S-adenosylhomocysteine
SAHH	S-adenosylhomocysteine hydrolase
SAM	S-adenosylmethionine
SASP	senescence-associated secretory phenotype
SIRT1	sirtuin 1
SIRT3	sirtuin 3
SIRT6	sirtuin 6
SIRT7	sirtuin 7
SLC7A11	solute carrier family 7 member 11
SOD2	superoxide dismutase 2
TNAP	tissue-nonspecific alkaline phosphatase
TNF-α	tumor necrosis factor alpha
TNFR1	tumor necrosis factor receptor 1
UPR	unfolded protein response
VSMC	vascular smooth muscle cell
WNT	Wingless-related integration site signaling pathway
