# Supplementary material for: The Big Five Functional Levels of Vascular Calcification
Source: Biology (Basel). 2026 Sep 25;15(19):1708. doi: 10.3390/biology15191708 (PMC13663498; doi:10.3390/biology15191708)
Supplement: Supplementary file 1 [file biology-15-01708-s001.zip › Table S4.pdf]

Table S4. Potential therapeutic and translational implications of the five functional levels of vascular calcification.

The table provides a translational interpretation of the framework rather than a list of established therapies. Candidate readouts are included where they are discussed or directly supported in the review; most are not validated as level-specific clinical biomarkers. The likely therapeutic window is conceptual and may differ according to disease context.

Functional level	Candidate therapeutic approaches	Potential translational readouts	Likely therapeutic window	Supporting evidence discussed in the review	Major translational limitations
**I. Mineral permissiveness**	Reduce excessive calcium-phosphate pressure; restore extracellular pyrophosphate (PPi); preserve ATP-ENPP1-PPi availability; limit excessive PPi degradation.	Serum calcium and phosphate; circulating PPi; indices of systemic mineral balance.	Before or during early mineral nucleation, particularly when systemic mineral dysregulation or loss of extracellular inhibition is prominent.	PPi replacement suppresses vascular mineralization in CKD and in severe genetic or metabolic susceptibility [21,42-44]. Manipulation of extracellular ATP/PPi metabolism alters calcification [24,45-50], and ATP-based treatment reduces calcification in progeroid mice [134].	Evidence is largely experimental. Circulating mineral variables do not necessarily reflect the local arterial microenvironment. These strategies mainly prevent or slow new deposition and have not been shown to remove mature vascular mineral.
**II. Mineral packaging, nucleation and lesion growth**	Delay calciprotein-particle maturation; reduce the mineralizing potential of extracellular vesicles; preserve inhibitory vesicle cargo; limit pathological matrix remodeling and elastolysis.	CPP maturation/composition; serum T50 as a measure of calcification propensity; EV phenotype; tissue mineral burden and mineral architecture, including dispersed microcalcification versus larger deposits.	Early nucleation and lesion growth, before extensive matrix incorporation and coalescence of mineral deposits.	Magnesium delays CPP-I to CPP-II maturation and reduces phosphate-induced calcification [64]. EV secretion and cargo influence mineralization [73,74,77]. Matrix retention, elastolysis and remodeling determine lesion growth [78,82-85]. Smaller hydroxyapatite particles and nanocrystals have strong inflammatory and cytotoxic effects [126,127].	CPP and EV measurements are method-sensitive, and serum T50 measures calcification propensity rather than circulating CPP concentration [69,70]. Mineral size and location cannot be inferred from total calcium burden alone. Relationships between microcalcification, macrocalcification and clinical plaque behavior remain context-dependent.
**III. Cellular reprogramming**	Limit maladaptive osteogenic reprogramming; modulate BMP-WNT signaling or other pathways that stabilize osteochondrogenic cell states.	Tissue RUNX2, BMP and WNT activity; osteogenic and chondrogenic markers in vascular cells.	During active lesion expansion when vascular-cell plasticity contributes to propagation of calcification.	VSMC-specific RUNX2 deletion reduces arterial calcification [22,95]. Pharmacological inhibition of BMP signaling reduces vascular calcification in hyperlipidemic and Mgp-deficient mice [99,100].	These pathways are pleiotropic and are not established level-specific therapeutic targets in patients. Osteogenic reprogramming may precede or follow mineral formation depending on disease context, and tissue markers are not validated clinical biomarkers.
**IV. Amplification and memory**	Reduce inflammatory and oxidative amplification; preserve mitochondrial function; interrupt persistent senescent, epigenetic or metabolic procalcific states.	Inflammatory mediators; oxidative-stress and mitochondrial readouts; senescence-associated and metabolic markers.	Established but still modifiable disease, particularly before self-reinforcing inflammatory, metabolic and senescent states become entrenched.	TNF-alpha neutralization reduces vascular BMP-WNT signaling and calcification in diabetic mice [102]. Antioxidant and mitochondrial interventions reduce calcification experimentally [128-135]. Manipulation of senescence-associated pathways also modifies calcification [139-146].	Most readouts are nonspecific and can lie both upstream and downstream of mineral formation. The relative importance of these pathways varies by disease and vascular compartment. Clinical evidence for reversing established calcification by targeting these mechanisms remains limited.
**V. Stress containment and homeostatic failure**	Preserve autophagic and proteostatic capacity; limit maladaptive ER-stress signaling; reduce regulated cell-death pathways when they contribute causally to mineralization.	Autophagic flux; unfolded-protein-response/CHOP signaling; pathway-specific markers of apoptosis, ferroptosis and other regulated cell-death programs.	Advanced cellular stress, with the aim of preventing further vesicle release, cell loss and generation of new nucleation substrates rather than removing existing mineral.	Manipulation of autophagy and ER-stress pathways alters vascular calcification [20,160-167]. Apoptosis provides mineralizing bodies [13], while ferroptotic susceptibility modifies calcification in experimental systems [172].	Adaptive and maladaptive stress responses can coexist, making timing critical. Cell-death markers are not equivalent to causal pathway activity. Inhibiting cellular failure may reduce further mineralization but does not by itself regress mature deposits.

Abbreviations: CPP, calciprotein particle; ENPP1, ectonucleotide pyrophosphatase/phosphodiesterase 1; EV, extracellular vesicle; PPi, inorganic pyrophosphate; T50, serum calcification-propensity assay; UPR, unfolded protein response. Candidate approaches and readouts are linked to the predominant functional role of each level; cross-level effects are expected. Most interventions discussed in the review have been tested for prevention or slowing of calcification, not for regression of established vascular mineral.
