# Supplementary material for: The Big Five Functional Levels of Vascular Calcification
Source: Biology (Basel). 2026 Sep 25;15(19):1708. doi: 10.3390/biology15191708 (PMC13663498; doi:10.3390/biology15191708)
Supplement: Supplementary file 1 [file biology-15-01708-s001.zip › Table S1.pdf]

Table S1. Historical Emergence and Mechanistic Consolidation of the Major Pathways of Vascular Calcification

Approximate year	Mechanism(s)	Conceptual milestone	Representative primary study
**1962**	**2. Failure of mineralization inhibitors — PPi**	Inorganic pyrophosphate was identified as a potent physiological inhibitor of calcium-phosphate precipitation, establishing the principle that extracellular fluids can remain supersaturated because mineral formation is actively restrained.	Fleisch H, Bisaz S. Mechanism of calcification: inhibitory role of pyrophosphate. *Nature*. 1962;195:911. PMID: **13893487**.
**1983**	**4. Extracellular and matrix vesicles**	Matrix-vesicle-like structures were identified within human atherosclerotic and medial arterial calcification, suggesting that membrane-bound structures could provide local sites for vascular mineral nucleation.	Tanimura A, McGregor DH, Anderson HC. Matrix vesicles in atherosclerotic calcification. *Proc Soc Exp Biol Med*. 1983;172:173–177. PMID: **6828462**.
**1993**	**7. BMP–WNT signaling — BMP origin**	BMP2 was detected in calcified human atherosclerotic lesions, providing early evidence that developmental osteogenic signaling participates in vascular mineralization.	Boström K, Watson KE, Horn S, et al. Bone morphogenetic protein expression in human atherosclerotic lesions. *J Clin Invest*. 1993;91:1800–1809. PMID: **8473518**.
**1997**	**2. Failure of mineralization inhibitors — MGP**	Deletion of *Mgp* caused spontaneous and extensive arterial calcification, providing genetic evidence that the normal vascular wall actively suppresses mineral deposition.	Luo G, Ducy P, McKee MD, et al. Spontaneous calcification of arteries and cartilage in mice lacking matrix GLA protein. *Nature*. 1997;386:78–81. PMID: **9052783**.
**1998**	**5. Extracellular matrix remodeling and degradation**	Extracellular matrix composition was shown to determine vascular-cell susceptibility to mineralization: collagen I and fibronectin promoted calcification, whereas collagen IV was inhibitory.	Watson KE, Parhami F, Shin V, Demer LL. Fibronectin and collagen I matrixes promote calcification of vascular cells in vitro, whereas collagen IV matrix is inhibitory. *Arterioscler*

			Thromb Vasc Biol. 1998;18:1964–1971. PMID: **9848891**.
**1999**	**6. Osteogenic reprogramming and VSMC plasticity**	Human medial calcification was shown to contain VSMCs associated with bone- and cartilage-related proteins, establishing that arterial mineralization is accompanied by an organized phenotypic response rather than simple passive precipitation.	Shanahan CM, Cary NR, Salisbury JR, et al. Medial localization of mineralization-regulating proteins in association with Mönckeberg's sclerosis. *Circulation*. 1999;100:2168–2176. PMID: **10571976**.
**2000**	**1. Elevated phosphate and calcium**	Elevated extracellular phosphate was shown to induce VSMC mineralization together with CBFA1/RUNX2 and osteocalcin, establishing mineral dysregulation as a direct experimental driver of vascular calcification.	Jono S, McKee MD, Murry CE, et al. Phosphate regulation of vascular smooth muscle cell calcification. *Circ Res*. 2000;87:E10–E17. PMID: **11009570**.
**2000**	**9. Inflammation**	TNF-α was shown to actively promote vascular-cell mineralization, moving inflammation from an associated feature of atherosclerosis to a mechanistic component of calcification.	Tintut Y, Patel J, Parhami F, Demer LL. Tumor necrosis factor-alpha promotes in vitro calcification of vascular cells via the cAMP pathway. *Circulation*. 2000;102:2636–2642. PMID: **11085968**.
**2000**	**14. Regulated cell death — apoptosis**	Apoptosis was shown to precede VSMC calcification; manipulating apoptosis changed mineral deposition, and apoptotic bodies themselves concentrated calcium and crystalline mineral.	Proudfoot D, Skepper JN, Hegyi L, et al. Apoptosis regulates human vascular calcification in vitro: evidence for initiation of vascular calcification by apoptotic bodies. *Circ Res*. 2000;87:1055–1062. PMID: **11090552**.
**2001**	**6. Osteogenic reprogramming and VSMC plasticity**	VSMC calcification was directly linked to loss of smooth-muscle lineage markers and induction of Cbfa1/RUNX2, establishing the modern concept of osteogenic phenotypic transition.	Steitz SA, Speer MY, Curinga G, et al. Smooth muscle cell phenotypic transition associated with calcification. *Circ Res*. 2001;89:1147–1154. PMID: **11739279**.

**2003**	**3. Calciprotein particles**	Fetuin-A was shown to bind nascent calcium phosphate and stabilize it as soluble mineral–protein colloids, establishing CPP formation as a physiological mineral-buffering mechanism.	Heiss A, DuChesne A, Denecke B, et al. Structural basis of calcification inhibition by alpha 2-HS glycoprotein/fetuin-A: formation of colloidal calciprotein particles. *J Biol Chem*. 2003;278:13333–13341. PMID: **12556469**.
**2003**	**2. Failure of mineralization inhibitors — ENPP1/PPi**	Loss-of-function mutations in *ENPP1* were identified as the cause of generalized arterial calcification of infancy, directly linking impaired extracellular PPi production to human arterial calcification.	Rutsch F, Ruf N, Vaingankar S, et al. Mutations in ENPP1 are associated with idiopathic infantile arterial calcification. *Nat Genet*. 2003;34:379–381. PMID: **12881724**.
**2004**	**2. Failure of mineralization inhibitors — vascular PPi**	Intact arterial tissue was shown to resist calcium-phosphate deposition through endogenous PPi; enzymatic PPi removal triggered hydroxyapatite formation, whereas exogenous PPi suppressed it.	Lomashvili KA, Cobbs S, Hennigar RA, Hardcastle KI, O'Neill WC. Phosphate-induced vascular calcification: role of pyrophosphate and osteopontin. *J Am Soc Nephrol*. 2004;15:1392–1401. PMID: **15153550**.
**2004**	**4. Extracellular and matrix vesicles — functional demonstration**	VSMCs exposed to altered calcium and phosphate were shown to generate both apoptotic bodies and matrix vesicles capable of nucleating basic calcium phosphate, moving vesicles from ultrastructural observation to experimental mechanism.	Reynolds JL, Joannides AJ, Skepper JN, et al. Human vascular smooth muscle cells undergo vesicle-mediated calcification in response to changes in extracellular calcium and phosphate concentrations. *J Am Soc Nephrol*. 2004;15:2857–2867. PMID: **15504939**.
**2005**	**7. BMP–WNT signaling — network consolidation**	MSX2 was shown to promote cardiovascular calcification through paracrine WNT signaling, linking earlier BMP observations to a broader developmental signaling network.	Shao JS, Cheng SL, Pingsterhaus JM, et al. Msx2 promotes cardiovascular calcification by activating paracrine Wnt signals. *J Clin Invest*. 2005;115:1210–1220. PMID: **15841209**.
**2008**	**10. Oxidative stress and mitochondrial dysfunction**	Oxidative stress was linked directly to osteogenic reprogramming through AKT-dependent modulation of RUNX2, establishing ROS as an active signal rather than simply a marker of vascular injury.	Byon CH, Javed A, Dai Q, et al. Oxidative stress induces vascular calcification through modulation of the osteogenic transcription factor Runx2 by AKT signaling. *J Biol Chem*. 2008;283:15319–15327. PMID: **18378684**.

**2009**	**11. Cellular senescence**	Replicatively senescent human VSMCs were shown to be markedly more susceptible to mineralization and to acquire an altered osteogenic and mineral-regulatory phenotype.	Nakano-Kurimoto R, Ikeda K, Uraoka M, et al. Replicative senescence of vascular smooth muscle cells enhances calcification through initiation of the osteoblastic transition. *Am J Physiol Heart Circ Physiol*. 2009;297:H1673–H1684. PMID: **19749165**.
**2009**	**6. Osteogenic reprogramming and VSMC plasticity — lineage proof**	Genetic fate mapping demonstrated that smooth-muscle-lineage cells generate osteochondrogenic precursor- and chondrocyte-like cells within calcifying arteries.	Speer MY, Yang HY, Brabb T, et al. Smooth muscle cells give rise to osteochondrogenic precursors and chondrocytes in calcifying arteries. *Circ Res*. 2009;104:733–741. PMID: **19197075**.
**2010**	**8. EndMT and alternative cellular sources — conceptual precedent**	Constitutively active ALK2 was shown to destabilize endothelial identity and generate mesenchymal, multipotent cells capable of osteogenic differentiation, establishing endothelial plasticity as a potential source of ectopic mineralizing cells.	Medici D, Shore EM, Lounev VY, et al. Conversion of vascular endothelial cells into multipotent stem-like cells. *Nat Med*. 2010;16:1400–1406. PMID: **21102460**.
**2012**	**13. Autophagy and ER stress — ER stress**	ATF4-dependent ER stress was shown to promote VSMC osteogenic differentiation and calcification in response to saturated fatty-acid stress.	Masuda M, Ting TC, Levi M, et al. Activating transcription factor 4 regulates stearate-induced vascular calcification. *J Lipid Res*. 2012;53:1543–1552. PMID: **22628618**.
**2013**	**8. EndMT and alternative cellular sources — vascular calcification**	Endothelial-lineage cells in *Mgp*-deficient and diabetic mice were shown to acquire stem/mesenchymal and subsequently osteogenic markers, connecting endothelial plasticity directly to vascular calcification.	Yao Y, Jumabay M, Ly A, et al. A role for the endothelium in vascular calcification. *Circ Res*. 2013;113:495–504. PMID: **23852538**.
**2013**	**13. Autophagy and ER stress — autophagy**	Autophagy was shown to counteract phosphate-induced calcification by limiting matrix-vesicle release, introducing the concept that stress responses can initially protect rather than promote mineralization.	Dai XY, Zhao MM, Cai Y, et al. Phosphate-induced autophagy counteracts vascular calcification by reducing matrix vesicle release. *Kidney Int*. 2013;83:1042–1051. PMID: **23364520**.

**2013**	**12. Epigenetic and metabolic reprogramming — epigenetic entry**	Pharmacological HDAC inhibition was shown to alter phosphate-induced VSMC calcification through alkaline phosphatase regulation, introducing chromatin regulation into the mechanistic landscape of vascular calcification.	Azechi T, Kanehira D, Kobayashi T, et al. Trichostatin A, an HDAC class I/II inhibitor, promotes Pi-induced vascular calcification via up-regulation of alkaline phosphatase. *J Atheroscler Thromb*. 2013;20:538–547. PMID: **23518467**.
**2013**	**10 ↔ 2. Mitochondrial dysfunction and loss of mineralization inhibitors**	In progeria, mitochondrial dysfunction reduced ATP availability while TNAP activity increased, converging on extracellular PPi deficiency. PPi replacement strongly reduced arterial calcification despite unchanged circulating calcium and phosphate, directly linking cellular bioenergetic failure to loss of extracellular mineral inhibition.	Villa-Bellosta R, Rivera-Torres J, Osorio FG, et al. Defective extracellular pyrophosphate metabolism promotes vascular calcification in a mouse model of Hutchinson–Gilford progeria syndrome that is ameliorated on pyrophosphate treatment. *Circulation*. 2013;127:2442–2451. PMID: **23690466**.
**2015**	**4. Extracellular and matrix vesicles — regulated secretion**	Calcifying VSMC vesicles were shown to arise through regulated multivesicular-body/exosomal trafficking, establishing vesicle biogenesis as an active cellular process rather than passive membrane shedding.	Kapustin AN, Chatrou MLL, Drozdov I, et al. Vascular smooth muscle cell calcification is mediated by regulated exosome secretion. *Circ Res*. 2015;116:1312–1323. PMID: **25711438**.
**2016**	**6. Osteogenic reprogramming — causal consolidation**	Smooth-muscle-specific *Runx2* deletion reduced vascular calcification without substantially altering plaque burden or systemic mineral metabolism, demonstrating a direct causal contribution of VSMC osteogenic reprogramming to lesion expansion.	Lin ME, Chen TM, Wallingford MC, et al. Runx2 deletion in smooth muscle cells inhibits vascular osteochondrogenesis and calcification but not atherosclerotic lesion formation. *Cardiovasc Res*. 2016;112:606–616. PMID: **27671804**.
**2022**	**14. Regulated cell death — ferroptosis**	Genetic and pharmacological manipulation of the SLC7A11–GSH–GPX4 axis showed that ferroptotic susceptibility causally modifies vascular calcification across VSMCs, arterial tissue and in vivo models, expanding regulated cell death beyond apoptosis.	Ye Y, Chen A, Li L, et al. Repression of the antiporter SLC7A11/glutathione/glutathione peroxidase 4 axis drives ferroptosis of vascular smooth muscle cells to facilitate vascular calcification. *Kidney Int*. 2022;102:1259–1275. PMID: **36063875**.

**2024**	**12. Epigenetic and metabolic reprogramming — metabolic–chromatin coupling**	Genetic manipulation of NR4A3 connected glycolytic flux and lactate production to histone lactylation and PHOSPHO1-dependent calcification, providing a direct mechanistic bridge between metabolism, chromatin and mineralization.	Ma W, Jia K, Cheng H, et al. Orphan nuclear receptor NR4A3 promotes vascular calcification via histone lactylation. *Circ Res*. 2024;134:1427–1447. PMID: **38629274**.
