# Supplementary material for: The Big Five Functional Levels of Vascular Calcification
Source: Biology (Basel). 2026 Sep 25;15(19):1708. doi: 10.3390/biology15191708 (PMC13663498; doi:10.3390/biology15191708)
Supplement: Supplementary file 1 [file biology-15-01708-s001.zip › Table S2.pdf]

Table S2. Disease context, vascular compartment and representative experimental models supporting the five-level framework.

Disease context	Predominant vascular compartment	Representative evidence and experimental models	Mechanistic emphasis within the five-level framework	Contextual interpretation
**Atherosclerosis**	Intimal (atherosclerotic plaque)	Human coronary/carotid atherosclerotic lesions; hyperlipidemic ApoE-/- and LDLR-/- mice; VSMC lineage tracing and smooth-muscle-specific Runx2 deletion; plaque and 3D models of EV-derived microcalcification [1,2,8,78,90,94,95,99].	Levels II-IV: EV retention and plaque microcalcification; VSMC plasticity and BMP-WNT signaling; inflammatory amplification.	Evidence primarily concerns calcification within an inflammatory, lipid-rich plaque. Relative mechanistic importance should not be assumed to be identical in medial calcification.
**Chronic kidney disease / uremia**	Predominantly medial; intimal and medial disease may coexist clinically	Human CKD/dialysis arteries and ex vivo arterial rings; uremic/CKD rodent models with mineral loading; CKD-derived CPP and EV studies; genetic/pharmacological manipulation of phosphate transport, pyrophosphate, oxidative stress and matrix remodeling [32,39,42,49,50,66,76,83,84,91,128,129,140,147].	All five levels are represented, with especially strong evidence for Level I mineral pressure/inhibitor failure, Level II CPP/EV/matrix effects, and Levels IV-V stress amplification and homeostatic failure.	CKD best illustrates convergence of systemic mineral dysregulation with local medial-wall responses. Severe experimental phosphate loading should be interpreted in relation to the more heterogeneous human CKD phenotype.
**Diabetes and metabolic disease**	Mixed: medial calcification is clinically prominent; several mechanistic mouse studies use an atherosclerotic/intimal background	Diabetic LDLR-/- mice; Ins2Akita/+ mice; insulin-resistant ob/ob mice; human VSMC studies of inflammatory and osteogenic signaling [19,101-103,107,109,160,162].	Levels III-IV, with contributions from Level V: BMP-MSX2-WNT signaling, EndMT/cellular plasticity, inflammation, metabolic stress, altered autophagy and vesicle-mediated signaling.	Compartment depends strongly on the model. Diabetic LDLR-/- mice combine metabolic disease with atherosclerosis and should not be generalized to isolated medial calcification.
**Monogenic loss of mineralization control**	Predominantly non-atherosclerotic arterial/medial calcification in the disorders and models discussed	Human ENPP1 loss-of-function causing generalized arterial calcification of infancy; Mgp-null mice; vascular transplantation and systemic ATP-pyrophosphate studies; Mgp-deficient lineage tracing and BMP inhibition [9,15,28,46-48,57,85,100].	Level I is dominant: loss of extracellular mineral inhibition. Cross-level effects extend to Level II matrix/mineral growth and Level III BMP-dependent cellular reprogramming.	Provides strong causal evidence for the pathways involved, but these disorders are not direct models of common acquired atherosclerotic or CKD-associated calcification.
**Progeroid / premature-aging models**	Predominantly non-atherosclerotic arterial calcification; medial involvement is emphasized in the experimental context	LmnaG609G/+ Hutchinson-Gilford progeria mice; interventions restoring pyrophosphate availability, extracellular ATP or mitochondrial function [21,134,135].	Level IV mitochondrial dysfunction/redox failure feeds back to Level I through reduced ATP-dependent pyrophosphate production; Level V stress failure also contributes.	Especially informative for causal coupling between bioenergetic failure and extracellular mineral inhibition, but the extreme premature-aging phenotype limits direct extrapolation to common disease.
**Marfan syndrome / primary extracellular-matrix disease**	Medial aortic microcalcification	Human Marfan aortas and Fbn1C1039G/+ mice; VSMC experiments with elastin-derived peptides and elastin-receptor/ERK signaling [87].	Level II matrix remodeling is central, with downstream Level III VSMC reprogramming and a possible Level IV structural-memory effect.	Shows how a primary matrix abnormality can lower the threshold for medial mineralization without implying the same initiating lesion in atherosclerosis.
**Monckeberg medial sclerosis**	Medial	Human calcified arteries and VSMCs isolated from medial lesions showing alkaline phosphatase, bone sialoprotein, osteocalcin and type II collagen [10].	Primarily Level III: direct human evidence for VSMC phenotypic change in medial calcification, interpreted within the Level I-II mineral/matrix context.	Establishes association with a medial osteochondrogenic phenotype but does not by itself determine the temporal order of mineral formation and cellular reprogramming.

Note. The compartment shown is the predominant setting represented by the cited evidence and does not imply anatomical exclusivity. Intimal and medial calcification can coexist, particularly in advanced systemic disease. The same mechanism may operate in both compartments with different relative importance. Valvular calcification is not included; where studies report both arterial and valvular findings, only the arterial evidence is used to support the vascular framework.
