# Supplementary material for: The Big Five Functional Levels of Vascular Calcification
Source: Biology (Basel). 2026 Sep 25;15(19):1708. doi: 10.3390/biology15191708 (PMC13663498; doi:10.3390/biology15191708)
Supplement: Supplementary file 1 [file biology-15-01708-s001.zip › Table S3.pdf]

Table S3. Functional assignment, cross-level interactions, supporting evidence and principal limitations of the 14 mechanisms included in the five-level framework.

Functional level and guiding question	Mechanism	Predominant causal role	Major cross-level effects	Evidence and main limitation
**Level I. Mineral permissiveness** *Can calcium-phosphate mineral form?*	**1. Elevated phosphate and calcium**	Increases extracellular calcium-phosphate supersaturation and favors mineral nucleation.	Mineral formation can subsequently induce cellular signaling and osteogenic responses.	Cell-free/fixed-cell, ex vivo and in vivo evidence; experimental ion concentrations do not establish temporal priority in all human disease.
	**2. Failure of mineralization inhibitors**	Removes extracellular restraints on crystal formation and growth.	PPi, MGP and fetuin-A also influence vesicle mineralization and mineral packaging.	Strong genetic, experimental and human evidence; relative contribution varies with disease context.
**Level II. Mineral packaging, nucleation and lesion growth** *Where does mineral nucleate and persist?*	**3. Calciprotein particles**	Packages nascent mineral and determines its maturation, persistence and cellular handling.	Mature CPPs activate inflammatory and oxidative responses.	Biochemical, cellular and CKD-associated evidence; circulating CPP measurements remain method-dependent.
	**4. Extracellular and matrix vesicles**	Provides membrane surfaces for local calcium-phosphate nucleation.	Vesicle composition depends on mineral inhibitors, cellular stress and cell death.	Human tissue, cellular and genetic evidence; EV populations are heterogeneous.
	**5. Extracellular matrix remodeling and degradation**	Creates and retains mineral-permissive surfaces and alters lesion growth.	Altered matrix can influence VSMC phenotype and provide persistent procalcific cues.	Human, genetic and experimental evidence; causal contribution varies with vascular compartment and model.
**Level III. Cellular reprogramming** *How do vascular cells respond?*	**6. Osteogenic reprogramming and VSMC plasticity**	Alters VSMC phenotype and promotes progression of established calcification.	Reprogrammed cells modify vesicles, matrix and mineral-regulatory pathways.	Lineage tracing and conditional genetic evidence; not required for the first mineral nucleus.
	**7. BMP-WNT signaling**	Stabilizes osteogenic and developmental signaling programs.	Interacts with inflammation, matrix signals and cellular plasticity.	Genetic and pharmacological evidence; pathway activation can occur before or after mineral formation.
	**8. EndMT and alternative cellular sources**	Expands the pool of mesenchymal/osteogenic cells within calcifying vessels.	Strongly influenced by inflammatory and BMP signaling.	Lineage-tracing and experimental evidence; contribution is model- and context-dependent.
**Level IV. Amplification and memory** *What amplifies and stabilizes the response?*	**9. Inflammation**	Amplifies cellular reprogramming and lesion progression.	Can precede mineralization or be induced by mineral particles.	Extensive experimental evidence; direction of causality varies with disease context.
	**10. Oxidative stress and mitochondrial dysfunction**	Reinforces procalcific signaling and metabolic dysfunction.	Can reduce ATP-dependent PPi production and promote cell-death pathways.	Genetic/pharmacological and disease-model evidence; ROS may be both upstream and downstream of mineralization.
	**11. Cellular senescence**	Stabilizes a persistent calcification-prone cellular state.	Alters inflammatory, matrix, redox and mineral-regulatory functions.	Cellular, genetic and aging-related evidence; senescence markers are individually nonspecific.
	**12. Epigenetic and metabolic reprogramming**	Maintains or modifies long-lived calcification-prone states.	Connects metabolism with osteogenic, inflammatory and stress-response pathways.	Functional manipulation of metabolic and epigenetic regulators; many associations remain context-specific.
**Level V. Stress containment and homeostatic failure** *Can cellular stress still be contained?*	**13. Autophagy and ER stress**	Determines whether cellular stress is successfully contained or becomes procalcific.	Alters vesicle release, proteostasis and cell-death susceptibility.	Genetic and pharmacological evidence across several models; adaptive and maladaptive effects depend on stress duration.
	**14. Regulated cell death**	Generates membrane-bound nucleation substrates when homeostasis fails.	Apoptotic bodies and ferroptotic injury feed mineral nucleation and inflammatory amplification.	Genetic/pharmacological and tissue evidence; individual death pathways are not universally required.

Note: Mechanisms are assigned according to their predominant causal role. Assignment is not exclusive, and individual mechanisms may influence other levels through feedback or secondary effects.
